# Bioinspired Optimization for Feature Selection in Post-Compliance Risk Prediction

**DOI:** 10.3390/biomimetics11030190

**Published:** 2026-03-05

**Authors:** Álex Paz, Broderick Crawford, Eric Monfroy, Eduardo Rodriguez-Tello, José Barrera-García, Felipe Cisternas-Caneo, Benjamín López Cortés, Yoslandy Lazo, Andrés Yáñez, Álvaro Peña Fritz, Ricardo Soto

**Affiliations:** 1 Escuela de Ingeniería en Construcción y Transporte, Pontificia Universidad Católica de Valparaíso, Avenida Brasil 2147, Valparaíso 2362804, Chile; alex.paz@pucv.cl (Á.P.); andres.yanez@pucv.cl (A.Y.); alvaro.pena@ucv.cl (Á.P.F.); 2 Laboratoire d’Étude et de Recherche en Informatique d’Angers (LERIA), Université d’ Angers, UFR Sciences, 2 Bd de Lavoisier, 49000 Angers, France; eric.monfroy@univ-angers.fr; 3 Escuela de Ingeniería Informática, Pontificia Universidad Católica de Valparaíso, Avenida Brasil 2241, Valparaíso 2362807, Chile; felipe.cisternas.c@mail.pucv.cl (F.C.-C.); benjamin.lopez.c@mail.pucv.cl (B.L.C.); yoslandy.lazo@pucv.cl (Y.L.); ricardo.soto@pucv.cl (R.S.); 4 Cinvestav Unidad Tamaulipas, Km. 5.5 Carretera Victoria-Soto La Marina, Victoria 87130, Tamaulipas, Mexico; ertello@cinvestav.mx; 5 Department of Computer Science, Universidad de Alcalá, Alcalá de Henares, 28805 Madrid, Spain

**Keywords:** bio-inspired optimization, swarm intelligence, metaheuristic algorithms, wrapper-based feature selection, engineering optimization, imbalanced classification, computational efficiency

## Abstract

Bio-inspired metaheuristic optimization offers flexible search mechanisms for high-dimensional predictive problems under operational constraints. In administrative risk prediction settings, class imbalance and feature redundancy challenge conventional learning pipelines. This study evaluates a wrapper-based metaheuristic feature selection framework for post-compliance income declaration prediction using real longitudinal administrative records. The proposed approach integrates swarm-inspired optimization with supervised classifiers under a weighted objective function jointly prioritizing minority-class recall and subset compactness. Robustness is assessed through 31 independent stochastic runs per configuration. The empirical results indicate that performance effects are learner-dependent. For variance-prone classifiers, substantial minority-class recall gains are observed, with recall increasing from 0.284 to 0.849 for k-nearest neighbors and from 0.471 to 0.932 for Random Forest under optimized configurations. For LightGBM, optimized models maintain high recall levels (0.935–0.943 on average) with low dispersion, suggesting representational stabilization and dimensional compression rather than large absolute recall improvements. Optimized subsets retain approximately 16–33 features on average from the original 76-variable space. Within the evaluated experimental protocol, the findings show that metaheuristic-driven wrapper feature selection can reshape predictive representations under class imbalance, enabling simultaneous control of minority-class performance and feature dimensionality. Formal institutional deployment and cross-domain generalization remain subjects for future investigation.

## 1. Introduction

Nature-inspired optimization algorithms constitute a central paradigm in contemporary engineering for addressing high-dimensional and nonlinear problems that are not amenable to analytical optimization. Bio-inspired and biomimetic metaheuristics abstract principles observed in natural systems—such as collective behavior, adaptation, competition, and self-organization—into population-based search mechanisms capable of navigating complex solution spaces. Swarm intelligence methods, evolutionary strategies, and population-driven learning dynamics translate biological processes into computational operators that balance exploration and exploitation under resource constraints. In engineering contexts, these algorithms provide flexible and scalable mechanisms for approximating near-optimal solutions in combinatorial and high-dimensional settings, where exhaustive search is infeasible [1,2,3,4].

From a biomimetic perspective, wrapper-based feature selection can be interpreted as an adaptive population process over a discrete combinatorial landscape. Candidate feature subsets evolve through stochastic interaction mechanisms inspired by biological systems, where selection pressure is imposed by task-specific fitness objectives. In this study, the biological abstraction is operationalized through swarm-based population dynamics that iteratively refine feature representations under imbalance-aware evaluation criteria. The bio-inspired component therefore functions as a structured search mechanism for navigating high-dimensional representational spaces under operational constraints, rather than as a purely heuristic enhancement.

In parallel, supervised machine learning models have become a standard tool for predictive analytics in operational and institutional environments. However, real-world administrative datasets typically deviate from the assumptions underlying benchmark datasets. They often exhibit heterogeneous feature spaces, strong class imbalance, temporal constraints, and limited tolerance for opaque or unstable model behavior. In such contexts, predictive performance is highly sensitive to feature relevance and redundancy, and models trained on high-dimensional representations may suffer from degraded generalization, reduced robustness, and limited interpretability [5,6,7].

Feature selection addresses these challenges by identifying compact subsets of informative variables that preserve predictive signal while reducing dimensionality and model complexity. Among the different feature selection paradigms, wrapper-based approaches are particularly effective in applied settings, as they directly evaluate candidate feature subsets through the performance of a downstream predictive model. This allows wrapper methods to capture interaction effects between variables, which are often critical in complex institutional datasets [8,9]. Nevertheless, wrapper-based feature selection induces a combinatorial optimization problem whose search space grows exponentially with the number of candidate features, rendering exhaustive exploration infeasible in nontrivial scenarios.

Metaheuristic algorithms provide a scalable mechanism to address this combinatorial challenge. Through population-based stochastic operators, they enable structured exploration of discrete feature subset spaces without imposing restrictive assumptions on feature independence or model structure. Consequently, metaheuristic-driven wrapper feature selection has been increasingly adopted in high-dimensional classification problems, demonstrating improvements in dimensional parsimony and predictive robustness [2,3,10].

Despite the growing literature on metaheuristic-driven feature selection, its behavior under real administrative constraints remains insufficiently characterized. Many empirical studies rely on curated benchmark datasets that do not reflect longitudinal irregularities, operational deadlines, and class imbalance typical of institutional systems. Furthermore, optimization is often evaluated using aggregate accuracy metrics, without explicit prioritization of minority-class detection or systematic assessment of variability across independent stochastic runs. As metaheuristic algorithms are inherently population-based and stochastic, stability and cross-run consistency become critical dimensions in applied decision-support environments [4,11]. These limitations motivate an experimental framework that explicitly integrates imbalance-aware objectives, repeated stochastic validation, and real administrative data.

In response to these considerations, the present study establishes a direct methodological alignment between identified limitations and experimental design. Specifically, it (i) operates on real longitudinal administrative records spanning 2012–2024 rather than synthetic benchmarks, (ii) formulates a weighted objective function based on minority-class recall and the number of selected features, rather than optimizing aggregate accuracy alone, and (iii) evaluates robustness through 31 independent stochastic optimization runs for the metaheuristic-based configurations under a stratified validation protocol. This design is structured to assess performance under realistic institutional conditions, with explicit attention to minority-class detectability, temporal coherence, and cross-run stability.

Importantly, this work does not seek to propose new data-level imbalance handling techniques such as resampling or synthetic instance generation. Following the taxonomy introduced by He and Garcia [5], imbalance mitigation strategies are commonly categorized into data-level and algorithm-level methods. Subsequent reviews highlight the growing role of feature-level and representation-oriented strategies, including feature selection and representation learning, as complementary mechanisms for handling imbalance without modifying the empirical class distribution [12,13]. The contribution of this study is positioned within this representational perspective: rather than altering the empirical class distribution or modifying classifier loss functions, the proposed framework optimizes the predictive feature subset under naturally imbalanced administrative conditions.

The proposed framework integrates metaheuristic feature selection with supervised classifiers representing different inductive biases, including distance-based, ensemble-based, and gradient-boosting models. Feature subsets are optimized under an objective function explicitly designed to prioritize minority-class recall, in line with the asymmetric costs associated with false negatives in this application. Model performance is evaluated using a repeated, stratified validation protocol, emphasizing predictive effectiveness, robustness across runs, and dimensional parsimony.

To evaluate these methodological design choices under realistic operational conditions, the empirical setting corresponds to a real-world, imbalanced binary classification problem derived from Chile’s income-contingent student loan system (FSCU). The dataset consists exclusively of longitudinal administrative records generated under regulatory compliance requirements, reflecting genuine institutional constraints rather than curated experimental conditions. This study does not include live production deployment or real-time institutional validation. Instead, it evaluates structural compatibility and predictive behavior under experimentally controlled conditions that mirror administrative constraints.

The main contributions of this work are summarized as follows:

- The formulation of a wrapper-based feature selection framework based on nature-inspired metaheuristic algorithms, applied to a real-world imbalanced classification problem under strict temporal constraints.
- An empirical evaluation of the impact of metaheuristic feature selection on minority-class detection, robustness, and stability across classifiers with different inductive biases.
- A characterization of the resulting feature subsets, highlighting trade-offs between predictive performance, dimensional reduction, and operational interpretability.
- An empirical examination of the structural conditions under which metaheuristic-driven wrapper feature selection can be decoupled from operational inference workflows in administrative decision-support environments, emphasizing offline optimization and batch prediction compatibility.

The remainder of this article is organized as follows. Section 2 reviews related work on nature-inspired optimization and metaheuristic-based feature selection. Section 3 describes the problem setting, data preparation, feature selection framework, and experimental protocol. Section 4 presents the experimental results, which are discussed in Section 5. Finally, Section 6 concludes the paper and outlines directions for future research.

## 2. Related Work

### 2.1. Nature-Inspired Optimization and Metaheuristic Algorithms

Nature-inspired metaheuristic algorithms have been extensively studied as general-purpose optimization techniques for complex engineering problems. Inspired by biological evolution, collective animal behavior, and natural processes, these algorithms use population-based search, stochastic operators, and adaptive mechanisms to balance exploration and exploitation in large, irregular search spaces. Foundational surveys and monographs have established the effectiveness of these methods across a broad range of continuous and discrete optimization problems, as well as their suitability for scenarios where gradient information is unavailable or unreliable [1,2,3,4]. From a biomimetic standpoint, these algorithms do not merely imitate biological systems metaphorically; they operationalize adaptive population dynamics as structured search mechanisms over complex landscapes, a property that becomes particularly relevant in combinatorial domains such as feature subset optimization.

Within this family, swarm intelligence algorithms and related population-based metaheuristics have gained particular attention due to their conceptual simplicity, scalability, and flexibility. Their applicability to engineering tasks such as parameter tuning, scheduling, resource allocation, and control has been widely documented [2,10]. However, many engineering applications involve decision variables defined in discrete or binary domains, requiring explicit adaptation mechanisms to bridge continuous search dynamics and combinatorial solution spaces. Several studies have addressed this challenge by proposing binarization strategies that enable continuous metaheuristics to operate effectively in binary search spaces [14].

### 2.2. Metaheuristic-Based Feature Selection in Supervised Learning

Feature selection has long been recognized as a critical preprocessing step in supervised learning, particularly in high-dimensional settings where irrelevant or redundant variables can impair model performance and interpretability. Classical taxonomies distinguish between filter, wrapper, and embedded approaches, each offering different trade-offs between computational cost and predictive effectiveness [8,9]. Among these paradigms, wrapper-based feature selection is especially attractive in applied contexts, as it directly optimizes feature subsets with respect to the performance of a specific predictive model.

The combinatorial nature of the feature selection problem renders exhaustive search infeasible for realistic datasets. To address this limitation, nature-inspired metaheuristic algorithms have been widely adopted as search strategies within wrapper-based frameworks. A comprehensive systematic review by Barrera-García et al. [15] documents the rapid expansion of metaheuristic-driven feature selection across domains, emphasizing their flexibility in problem formulation, objective design, and classifier integration. Consistent with this review, multiple empirical studies report improvements in predictive performance and dimensional parsimony when metaheuristic optimization guides subset selection [2,3,10].

Nevertheless, prior work also indicates that the effectiveness of metaheuristic-based feature selection is highly sensitive to design choices, including the objective function, evaluation protocol, and performance metrics. In particular, many studies rely on aggregate accuracy measures that may be inadequate for imbalanced classification problems. Furthermore, relatively limited attention has been paid to the robustness of feature selection outcomes across repeated runs, despite the inherent stochasticity of metaheuristic search processes [4,11].

However, relatively few studies explicitly formulate minority-class–oriented objectives within wrapper-based metaheuristic feature selection, particularly in real administrative datasets subject to temporal, regulatory, and governance constraints. In many applied works, imbalance is treated implicitly through global performance metrics rather than through objective functions that directly prioritize minority detection. This gap suggests that further empirical analysis is required to examine how objective function design interacts with stochastic search dynamics in imbalanced institutional settings, especially when evaluation protocols incorporate repeated independent runs to assess robustness.

### 2.3. Predictive Modeling Under Class Imbalance and Operational Constraints

Imbalanced classification problems are pervasive in applied engineering and institutional domains, especially in risk assessment, compliance monitoring, and anomaly detection. In these contexts, the minority class often corresponds to events of primary operational interest, while standard learning algorithms tend to be biased toward the majority class. As a consequence, accuracy-based evaluation can obscure poor performance on the minority class and lead to models that are unsuitable for decision support [5,11]. A substantial portion of the imbalance literature has focused on algorithm-level adaptations and ensemble-based strategies, including bagging-, boosting-, and hybrid-based approaches designed to rebalance predictive behavior without necessarily modifying feature representations [16]. While these methods have demonstrated effectiveness in many domains, they predominantly operate at the classifier or aggregation level rather than explicitly addressing the structure of the predictive feature space.

Data-level techniques such as oversampling, undersampling, and synthetic instance generation have been widely studied in the imbalance literature [5]. In addition to data-level and algorithm-level adaptations, subsequent research has increasingly considered feature-level and representation-oriented strategies as complementary mechanisms for handling imbalance without altering the empirical class distribution [12,13]. In institutional compliance settings, preserving the original class distribution is often desirable to maintain traceability and auditability. Accordingly, this study focuses on representation-oriented optimization through metaheuristic-driven feature subset selection, rather than modifying the underlying data distribution.

Recent research has increasingly emphasized the integration of machine learning and metaheuristic optimization techniques for individual-level risk assessment in real-world systems. A systematic literature review by Paz et al. [17] highlights the growing adoption of hybrid approaches combining supervised learning models with metaheuristic optimization, particularly for credit risk and compliance-related applications. The review also identifies persistent challenges related to class imbalance, feature relevance, and the lack of robustness analysis in applied studies.

In addition to class imbalance, real administrative datasets introduce further operational constraints, including heterogeneous data types, temporal dependencies, and strict requirements on interpretability and reproducibility. Many existing studies still evaluate metaheuristic-based feature selection on static datasets without enforcing temporal coherence or explicitly analyzing stability across repeated optimization runs. This limits the transferability of methodological advances to real-world institutional decision-support systems.

Taken together, the literature reveals a structural gap at the intersection of three dimensions: (i) the use of real longitudinal administrative datasets, (ii) explicit minority-class–oriented optimization objectives within wrapper-based metaheuristics, and (iii) systematic robustness evaluation across independent stochastic runs. Existing studies typically address these aspects in isolation rather than jointly. Addressing them simultaneously is essential to evaluate whether imbalance-aware metaheuristic feature selection can yield stable and operationally interpretable representations in institutional compliance settings. The present study investigates this intersection within the context of Chile’s income-contingent student loan system.

## 3. Methodological Framework and Experimental Design

### 3.1. Problem Definition and Prediction Scope

This study addresses a binary classification problem arising from the operation of an income-contingent student loan system, in which beneficiaries are required to periodically submit income declarations to determine their repayment obligations. The predictive task involves identifying borrowers likely to discontinue mandatory income declaration submissions (hereafter referred to as non-compliance), as opposed to those who remain compliant with the declaration process.

Formally, let $D={\left\{\left(x_{i},y_{i}\right)\right\}}_{i=1}^{N}$ denotes the dataset, where $x_{i}\in R^{p}$ represents the *p*-dimensional feature vector associated with borrower *i*, constructed exclusively from information available up to the borrower’s last observed income declaration. The binary target variable $y_{i}\in \left\{0,1\right\}$ indicates declaration behavior in the subsequent period, where $y_{i}=1$ denotes income declaration discontinuation (i.e., the borrower fails to submit any further mandatory income declarations after the reference point), and $y_{i}=0$ denotes continued compliance with the declaration process. The resulting classification problem is inherently imbalanced, as the proportion of borrowers who discontinue declarations is substantially smaller than that of compliant borrowers.

The prediction task is formulated under strict temporal constraints. For each borrower, features are constructed exclusively from information available up to the last observed income declaration. No variables derived from future behavior or post-discontinuation outcomes are included. This design enforces temporal coherence and prevents information leakage, ensuring that predictive models operate under realistic deployment conditions.

The scope of the analysis is further restricted to borrowers who have submitted at least one income declaration prior to the observation window. This restriction reflects the operational objective of identifying potential discontinuation among active participants in the system, rather than modeling initial non-compliance. Consequently, the task focuses on detecting early signals of disengagement within an ongoing administrative process.

From an institutional perspective, false negatives—borrowers who discontinue declarations but are incorrectly classified as compliant—entail higher operational risk than false positives. Accordingly, model evaluation and optimization emphasize performance on the minority class, particularly recall. This asymmetric cost structure directly informs the design of the feature selection objective function and the choice of evaluation metrics, as detailed in subsequent sections.

### 3.2. Data Source and Cohort Construction

The data used in this study were obtained from administrative records of an income-contingent student loan system managed by a Chilean higher education institution. The database integrates longitudinal, borrower-level information generated during the routine operation of the loan program, including academic trajectories, financial attributes, and income declaration behavior. All records correspond to real operational data collected for administrative purposes, not for research-driven data acquisition.

The underlying data infrastructure is organized as a relational database, in which each borrower is uniquely identified and consistently linked across multiple tables. This structure enables the reconstruction of individual borrower histories while preserving the temporal ordering of academic, financial, and declaration-related events.

**Figure 1:**
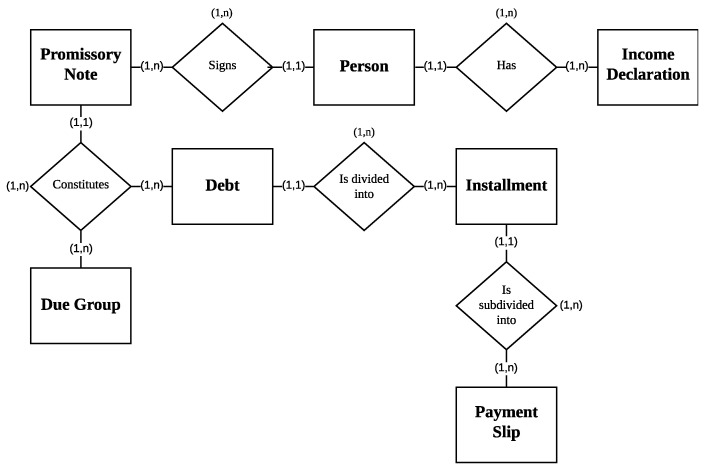
Relational schema of the administrative database.

Figure 1 illustrates the relational schema of the administrative database, highlighting the main entities involved and their relationships. This schema supports the integration of heterogeneous institutional data sources required for cohort definition and subsequent predictive modeling.

The analytical cohort was defined through a set of inclusion and exclusion criteria designed to ensure temporal coherence and operational relevance. Only borrowers with loan obligations from 2012 onward were considered to ensure consistency with the system’s current operational framework. In addition, borrowers were required to have submitted at least one income declaration prior to the prediction reference point, as the objective of the study is to identify declaration discontinuation among active participants rather than to model initial non-compliance.

Borrowers for whom a unique identifier or the temporal information required to define the prediction reference point could not be reliably established were excluded from the cohort. After applying these criteria, a final population suitable for predictive analysis was obtained.

Table 1 summarizes the cohort construction process, reporting the number of remaining and excluded records at each filtering stage.

For each borrower included in the final cohort, a single analytical observation was defined based on the last observed income declaration. All explanatory information used in subsequent modeling stages was restricted to data available up to this reference point, while the target variable captured whether the borrower subsequently discontinued the declaration process. This design enforces temporal coherence and reflects realistic deployment conditions for predictive decision support.

Table 2 reports the main descriptive characteristics of the resulting dataset, including cohort size, class distribution, and feature space composition. These characteristics highlight the imbalanced nature of the classification problem addressed in this study.

This cohort definition establishes a temporally coherent and operationally relevant population, which serves as the basis for subsequent feature construction and preprocessing steps.

### 3.3. Feature Construction and Initial Feature Space

Feature construction was guided by the objective of representing borrower behavior and conditions using information that is operationally available and temporally coherent with the defined prediction point. The initial feature space integrates heterogeneous variables derived from academic records, financial attributes, and income declaration histories, capturing the multidimensional nature of borrower engagement within the loan system.

All features were constructed at the individual borrower level and computed exclusively from information available up to the last observed income declaration. No variables derived from post-discontinuation events or future outcomes were included. This constraint ensures consistency with the prediction scope defined in Section 3.1 and prevents information leakage during model training and evaluation.

The resulting feature space combines demographic, academic, financial, and declaration-derived attributes. In addition to static institutional characteristics, this stage incorporates variables summarizing historical income declaration behavior, including counts, averages, temporal differences, and variability measures. Together, these variables provide a comprehensive representation of borrower status, financial exposure, and behavioral dynamics prior to the prediction reference point.

A detailed overview of the initial feature space, including variable names, data types, and observed value ranges, is reported in Table 3. Concise semantic descriptions of each variable are provided in Table 4.

The resulting initial feature space is intentionally high-dimensional, reflecting the richness of the available administrative data. While this representation preserves potentially informative signals, it also introduces redundancy and correlated information that may hinder predictive performance and model stability. These characteristics motivate the subsequent preprocessing and metaheuristic-based feature selection stages described in the following sections.

Table 5 summarizes the dimensionality of the initial feature space by semantic feature group prior to preprocessing.

### 3.4. Data Cleaning and Preprocessing

Data cleaning and preprocessing were performed to ensure internal consistency, temporal coherence, and compatibility with wrapper-based feature selection and supervised learning. Given the administrative and longitudinal nature of the records, all transformations were designed to preserve institutional meaning while reducing noise, sparsity, and redundancy in the feature space. A strict temporal constraint was enforced throughout the preprocessing pipeline to prevent information leakage, ensuring that all features were derived exclusively from information available up to the borrower-specific reference time. The outcome of this process is a clean, temporally coherent, and model-ready dataset suitable for subsequent optimization and predictive modeling.

#### 3.4.1. Consistency and Missing Data

An initial consistency assessment was conducted across the full feature space to identify structural anomalies, invalid values, and systematic missingness patterns. Missing data were evaluated at the feature level to distinguish between variables affected by sporadic absence and those exhibiting structural incompleteness. Features presenting excessive missingness or lacking operational interpretability were excluded from further analysis, as they provide limited and potentially misleading information.

For the remaining variables, missing values were handled using conservative, variable-specific strategies consistent with their semantic meaning and data type, including constant-value encoding and category-preserving treatments where appropriate. This approach avoids aggressive imputation schemes that could introduce artificial patterns or temporal leakage, while preserving the informative variability inherent to real-world administrative data. A detailed breakdown of missingness patterns across features is provided in Appendix A.1.

#### 3.4.2. Categorical Representation

Categorical variables were examined to mitigate instability caused by rare levels, invariant categories, or overly fragmented representations. Categories with negligible frequency were consolidated when semantically appropriate, and invariant variables were removed, as they do not contribute discriminative information. These steps reduce unnecessary sparsity in the encoded feature space and improve model robustness.

After consolidation, categorical variables were transformed using one-hot encoding to obtain a numerical representation compatible with the selected learning algorithms. This encoding strategy preserves categorical semantics while enabling efficient integration with numerical features during optimization and model training. Representative frequency distributions of categorical variables prior to consolidation are reported in Appendix A.2.

#### 3.4.3. Numerical Screening and Redundancy Control

Numerical variables were screened to detect degenerate cases, including near-constant features and variables exhibiting implausible or non-informative value ranges. Such variables were removed to prevent numerical instability and reduce noise during learning.

**Figure 2:**
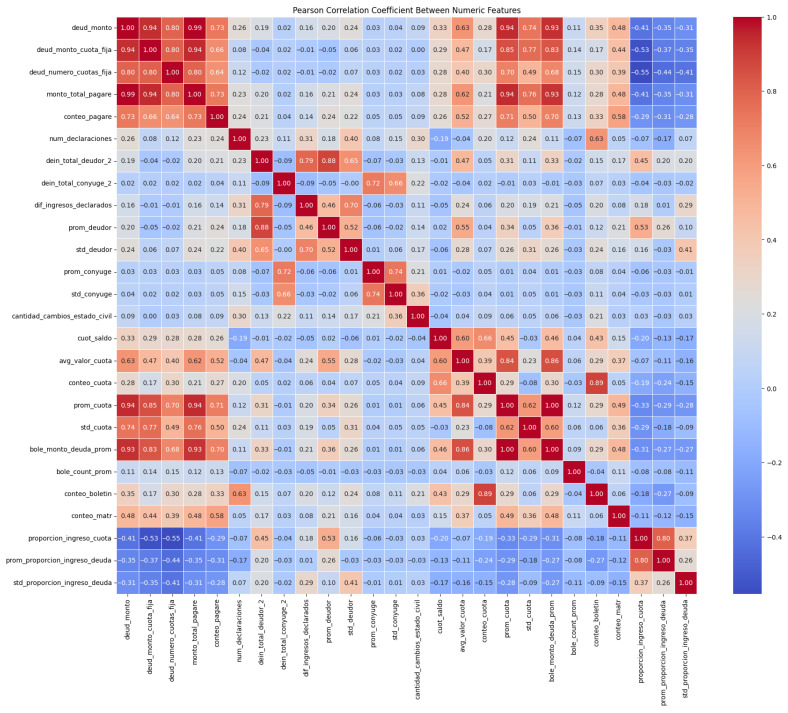
Pearson correlation matrix of numerical features prior to redundancy control.

To control redundancy in the numerical feature space, pairwise correlations were analyzed using the Pearson correlation coefficient. Figure 2 illustrates the correlation structure prior to redundancy filtering. Highly correlated feature pairs were identified, and redundant variables were removed based on operational interpretability and representational relevance. This step reduces multicollinearity, improves numerical conditioning, and contributes to a more compact and stable feature space prior to wrapper-based feature selection. Detailed distributions of numerical variables prior to screening are reported in Appendix A.3.

#### 3.4.4. Date Processing

Date-related variables were converted to numeric representations compatible with machine learning models while preserving temporal coherence within the defined prediction scope. Raw date fields were processed to extract structured temporal information, such as calendar components or elapsed-time representations, anchored to a borrower-specific reference point.

Throughout this process, strict temporal separation between feature construction and outcome observation was enforced. No date-derived information extending beyond the reference time was incorporated, thereby preventing information leakage and ensuring that all predictors reflect information realistically available at deployment time. Descriptive analyses of date-derived variables and their distributions are reported in Appendix A.4.

#### 3.4.5. Scaling

To ensure numerical comparability across features and stable numerical behavior during optimization and model training, all numerical variables were standardized using z-score normalization. Each numerical feature was transformed as:

(1) $$z=\frac{x-\mu }{\sigma },$$

where μ and σ denote the mean and standard deviation estimated exclusively from the training data and subsequently applied to the validation and test sets.

This scaling procedure prevents features with larger numeric ranges from disproportionately influencing the learning process and helps ensure numerically stable, well-conditioned optimization for both wrapper-based feature selection and supervised learning algorithms.

#### 3.4.6. Final Preprocessed Dataset

After completing the data cleaning and preprocessing pipeline, a final dataset suitable for wrapper-based feature selection and supervised learning was obtained. This dataset integrates the cumulative effects of consistency checks, handling of missing data, categorical encoding, numerical screening, redundancy control, date processing, and feature scaling, while preserving strict temporal coherence within the defined prediction scope.

Table 6 summarizes the main characteristics of the preprocessed dataset, including the number of observations and the dimensionality of the resulting feature space by feature type. The increase in dimensionality is primarily driven by the expansion of categorical variables through one-hot encoding. This table provides a concise overview of the modeling input without duplicating the detailed variable-level descriptions reported in previous sections.

For completeness and reproducibility, a detailed listing of the final feature space used in the modeling pipeline is provided in Appendix A.5. This appendix reports the full set of 76 variables retained after preprocessing, indexed consistently with the feature identifiers used throughout the experimental analysis and the heatmap-based feature selection results.

### 3.5. Metaheuristic-Based Feature Selection Framework

This study addresses feature selection as a wrapper-based optimization problem, where the objective is to identify compact and informative subsets of variables that maximize predictive performance under realistic deployment constraints. Given the high dimensionality of the preprocessed feature space and the combinatorial nature of the task, the selection process is formulated as a binary optimization problem and solved using population-based metaheuristic algorithms.

#### 3.5.1. Search Space and Solution Encoding

Let *p* denote the total number of candidate features available after data cleaning and preprocessing. Each candidate solution is represented as a binary vector

$$s=(s_{1},s_{2},\ldots ,s_{p}),$$

where $s_{j}=1$ indicates that the *j*-th feature is included in the selected subset, and $s_{j}=0$ indicates that it is excluded. This representation defines a discrete search space of size $2^{p}$, which grows exponentially with the number of available features “*p*” and makes exhaustive search computationally infeasible for realistic datasets.

The binary encoding establishes a direct one-to-one correspondence between the dimensions of the optimization space and the features in the dataset, ensuring interpretability of the resulting solutions. Each binary vector uniquely defines a feature subset that can be evaluated using a supervised learning model under a wrapper-based selection strategy.

To enable the application of population-based metaheuristic algorithms originally formulated for continuous search spaces, candidate solutions are internally represented as continuous-valued vectors. These continuous representations are subsequently mapped to binary feature selection vectors via a binarization mechanism that converts real-valued positions into binary inclusion decisions. This approach allows continuous metaheuristics to explore the discrete feature selection space while preserving their intrinsic search dynamics.

The adopted encoding strategy provides a flexible and algorithm-independent interface between the optimization process and the predictive modeling pipeline. By maintaining a consistent binary representation across all metaheuristic algorithms, the framework ensures comparability of selected feature subsets and facilitates systematic analysis of feature selection behavior across repeated optimization runs.

#### 3.5.2. Objective Function Definition

The wrapper-based feature selection problem is formulated as an optimization task in which the quality of each candidate feature subset is evaluated through the predictive performance of a supervised learning model. Rather than relying on surrogate statistical criteria, the objective function is defined to directly reflect the operational goals of the application.

Given the risk-sensitive nature of the prediction task and the severe impact of false negatives, the optimization process is explicitly guided by minority-class recall. Optimizing ${\mathrm{Recall}}_{\min}$ directly minimizes the number of non-compliant borrowers that remain undetected, aligning the feature selection process with institutional risk-management priorities.

Let *n* denote the total number of candidate features after preprocessing, and let $X\in {\left\{0,1\right\}}^{n}$ be a binary decision vector representing a candidate feature subset, where

(2) $$x_{i}=\left\{\begin{array}{cc} 1, & \mathrm{if}\mathrm{the}i\mathrm{-th}\mathrm{feature}\mathrm{is}\mathrm{selected},\\ 0, & \mathrm{otherwise}, \end{array}\right.\;i=1,\ldots ,n.$$

The objective function is defined as

(3) $$\min f\left(X\right)=1-{\mathrm{Recall}}_{\min}\left(X\right),$$

subject to

(4) $$\sum_{i=1}^{n}x_{i}\geq 1,$$

where ${\mathrm{Recall}}_{\min}\left(X\right)$ denotes the minority-class recall obtained by training and evaluating the classifier using the feature subset *X*.

This formulation implicitly balances predictive performance and subset compactness, as overly large feature subsets tend to reduce generalization performance and increase variability across optimization runs.

#### 3.5.3. Optimization Strategy

The feature selection problem defined in the previous sections is addressed using a wrapper-based optimization strategy driven by population-based, nature-inspired metaheuristic algorithms [15]. Given the exponential size of the binary search space and the impracticality of exhaustive exploration, metaheuristics provide an effective way to navigate large combinatorial spaces within constrained computational budgets.

In this study, three widely adopted bio-inspired metaheuristic algorithms are considered: Particle Swarm Optimization (PSO), Grey Wolf Optimizer (GWO), and Whale Optimization Algorithm (WOA). The selection of these algorithms follows three explicit methodological criteria: (i) compatibility with binary wrapper feature selection, (ii) computational efficiency under repeated stochastic evaluation, and (iii) empirical prevalence in feature selection literature, including imbalanced classification scenarios.

First, the feature selection task is formulated as a binary combinatorial optimization problem in which candidate solutions represent subsets of selected features. PSO, GWO, and WOA have well-established binary adaptations through transfer functions that map continuous position updates into probabilistic binary decisions ([18,19,20]). These binary variants are extensively documented in wrapper-based feature selection research. While other metaheuristics such as Genetic Algorithm (GA) and Ant Colony Optimization (ACO) are also applicable to combinatorial optimization, the selected algorithms provide direct and computationally efficient binary formulations without requiring crossover operators or pheromone matrix management.

Second, the experimental protocol involves 31 independent optimization runs per metaheuristic–classifier configuration. Under this repeated stochastic design, computational efficiency becomes a central consideration. PSO, GWO, and WOA are population-based yet structurally lightweight algorithms characterized by simple position-update equations and limited parameterization [18]. In contrast, GA incorporates crossover and mutation operators that introduce additional generational processing steps [21], while ACO relies on pheromone matrix construction and updating mechanisms that maintain and reinforce solution memory across iterations [22]. Under repeated multi-run evaluation in medium-dimensional search spaces, such structural differences may influence overall computational burden. The selected algorithms therefore provide a balanced trade-off between exploration capacity and computational tractability under the adopted validation design.

Third, PSO and GWO are among the most recurrent population-based metaheuristics reported in wrapper-based feature selection studies. WOA, although less frequent than PSO or GWO in the reviewed corpus, appears within the group of recurrent bio-inspired optimization methods applied to feature selection problems. Its inclusion allows evaluating a representative and comparatively recent search mechanism alongside more established algorithms [15].

PSO is inspired by social learning mechanisms observed in natural swarms and balances exploration and exploitation through the interaction between individual and collective experience [23,24]. GWO models a leadership hierarchy and cooperative hunting strategy, enabling efficient convergence with a limited number of control parameters [19,25,26]. WOA simulates the bubble-net hunting behavior of humpback whales and alternates between exploration and exploitation through encircling, spiral updating, and random search mechanisms [27,28].

All metaheuristic algorithms operate under a common wrapper-based optimization framework characterized by the binary solution encoding described in Section 3.5.1 and the objective function defined in Section 3.5.2. At each iteration, a population of candidate solutions is maintained, where each solution corresponds to a binary feature subset obtained by applying a binarization mechanism to the algorithm’s internal representation. Candidate subsets are evaluated by training a supervised learning model and computing the corresponding objective value.

To ensure methodological consistency and fair comparison across algorithms, all metaheuristics are executed under identical wrapper conditions. The same solution encoding, objective function, and evaluation protocol are used uniformly, and no algorithm-specific adaptive control or parameter-tuning mechanisms are introduced within the optimization loop. This design isolates the effect of the metaheuristic search strategy itself, allowing performance differences to be attributed to intrinsic algorithmic dynamics rather than auxiliary adaptation schemes.

The outcome of the optimization process is a collection of feature subsets obtained across multiple independent runs, reflecting both the stochastic nature of metaheuristic search and the robustness of the selected features. These subsets serve as the basis for evaluating predictive performance and stability.

### 3.6. Evaluation Metrics

The evaluation of feature subsets selected by the metaheuristic optimization process is conducted using classification metrics derived from the confusion matrix. While the optimization procedure is explicitly driven by minority-class performance, a broader set of metrics is reported to enable a comprehensive assessment of classifier behavior and potential trade-offs. This emphasis is consistent with risk-sensitive decision-making and common practice in imbalanced classification settings [5].

Let the minority (event) class be denoted as y=1 and the majority (non-event) class as y=0. For a set of predictions $\hat{y}$, we define:

- TP (true positives): number of instances with y=1 and $\hat{y}=1$.
- FN (false negatives): number of instances with y=1 and $\hat{y}=0$.
- FP (false positives): number of instances with y=0 and $\hat{y}=1$.
- TN (true negatives): number of instances with y=0 and $\hat{y}=0$.

In the context of the studied problem, false negatives are borrowers who discontinue income declaration but are incorrectly classified as compliant, which is the most critical error from an operational perspective [29].

Minority-class recall quantifies the ability of a model to correctly identify non-compliant borrowers among all true non-compliant cases. Because it directly penalizes false negatives, minority-class recall is adopted as the primary performance indicator and is defined as:

(5) $${\mathrm{Recall}}_{\min}=\frac{\mathrm{TP}}{\mathrm{TP}+\mathrm{FN}}.$$

Minority-class precision complements recall by measuring the reliability of positive predictions, indicating the proportion of predicted non-compliant borrowers that are indeed non-compliant. This metric is particularly relevant in operational settings where false positives may trigger unnecessary administrative actions or resource-intensive interventions:

(6) $${\mathrm{Precision}}_{\min}=\frac{\mathrm{TP}}{\mathrm{TP}+\mathrm{FP}}.$$

To summarize the trade-off between detection capability and prediction reliability, the minority-class F1-score is reported as the harmonic mean of precision and recall:

(7) $$F1_{\min}=2\cdot \frac{{\mathrm{Precision}}_{\min}\cdot {\mathrm{Recall}}_{\min}}{{\mathrm{Precision}}_{\min}+{\mathrm{Recall}}_{\min}}.$$

Although the feature selection process is guided by minority-class performance, the same metrics are also computed for the majority class by reversing the positive label (i.e., treating y=0 as the event). This complementary reporting supports a more complete characterization of classifier behavior, facilitates interpretation of confusion matrices, and enables the identification of trade-offs introduced by minority-focused optimization.

### 3.7. Predictive Models

The predictive impact of the feature subsets selected by the metaheuristic optimization framework is evaluated using three supervised classification models commonly employed in imbalanced binary classification problems: k-Nearest Neighbors (KNN), Light Gradient Boosting Machine (LightGBM), and Random Forest (RF). These models represent complementary inductive biases and levels of model complexity, enabling a robust assessment of how wrapper-based feature selection interacts with different learning mechanisms.

KNN is included as a distance-based, non-parametric classifier whose predictions rely directly on feature-space similarity. As a result, KNN is particularly sensitive to feature relevance, scaling, and redundancy, making it a suitable baseline for assessing whether metaheuristic-driven feature selection improves discriminative structure by removing noisy or redundant variables.

Gradient-boosted decision trees are represented by LightGBM, which constructs trees sequentially to exploit complex feature interactions and fine-grained decision boundaries. LightGBM is particularly effective in high-dimensional and imbalanced settings and serves as a strong reference model for evaluating whether feature selection contributes to robustness, stability, or parsimony when combined with boosting-based ensembles.

Tree-based ensemble learning is represented by Random Forest, which aggregates multiple decision trees trained on bootstrap samples and randomized feature subsets. RF is known for its robustness to noise, its ability to model non-linear interactions, and its inherent feature subsampling mechanism. Its inclusion enables assessing whether explicit feature selection provides benefits beyond those already induced by ensemble-based randomness.

### 3.8. Experimental Design and Validation Strategy

The experimental design is structured to ensure methodological consistency, fair comparison across metaheuristic algorithms, and reproducibility of results. All feature selection experiments are conducted under a unified validation strategy and comparable computational budgets.

The dataset is partitioned into training and testing subsets using a stratified splitting strategy that preserves the class distribution of the target variable. Feature selection is performed exclusively on the training data within each experimental run, and the resulting feature subsets are applied without modification to both training and testing partitions, ensuring strict separation between optimization and performance assessment and preventing information leakage.

To control computational effort and ensure comparability across optimization strategies, all metaheuristic algorithms are executed using a fixed population size of 10 and a maximum of 100 iterations. These parameters are kept constant across all experiments to isolate the effect of the feature selection process from differences in search budget.

All supervised learning models are trained using their default hyperparameter configurations as provided by the corresponding libraries. No additional hyperparameter tuning is performed, in order to isolate the effect of metaheuristic-driven feature selection from potential gains attributable to classifier optimization.

To account for the stochastic nature of metaheuristic search, each experimental configuration is executed multiple times using different random seeds (31 independent runs per algorithm). Predictive performance is computed independently for each run and subsequently aggregated, enabling the analysis of robustness and variability across executions.

This design ensures that observed differences in predictive behavior are attributable to the feature selection process itself, while maintaining strict control over computational effort and experimental consistency.

Although the population size and iteration count were fixed to ensure controlled and comparable computational budgets across algorithms, we acknowledge that metaheuristic performance can be sensitive to parameterization. The adopted configuration (population size = 10, maximum iterations = 100) was selected to provide a balanced trade-off between exploration capacity and computational tractability under a repeated 31-run stochastic design. A dedicated parameter sensitivity analysis could further investigate the effect of alternative population sizes and iteration counts on minority-class recall and subset compactness. However, the objective of this study is to isolate the impact of wrapper-based feature selection under standardized search conditions rather than to optimize each metaheuristic’s internal configuration. Parameter sensitivity analysis is therefore identified as a direction for future research.

### 3.9. Implementation Details

All experiments were implemented in Python (version 3.11.9) using a unified and reproducible computational pipeline. Data preprocessing, feature engineering, and dataset construction were carried out using standard scientific computing libraries to ensure consistent handling of numerical and categorical variables throughout the workflow.

The experimentation was executed in a private server equipped with an Intel Core i9-10900K CPU and 64 gigabytes of RAM. The dataset was divided into a training set and an independent test set in proportion of 80% and 20% respectively, using a stratifying strategy to ensure that the distribution of the target variable remains intact in both dataset.

Metaheuristic-based feature selection was implemented using the MealPy library [30] (version 3.0.3), which provides standardized implementations of population-based optimization algorithms. The PSO, GWO, and WOA algorithms were instantiated using their “Original” variants provided by the framework. For all metaheuristics, the population size was fixed at 10 individuals and the number of epochs (iterations) was set to 100. No additional algorithm-specific parameter tuning was performed; all remaining hyperparameters were kept at their default values as defined in the MealPy implementation to ensure methodological consistency and avoid optimization bias across algorithms.

Custom wrapper routines were developed to interface the metaheuristic search process with the supervised learning models, enforcing the experimental protocol defined in Section 3.8. Supervised learning models were implemented using widely adopted machine learning libraries, namely scikit-learn (version 1.7.1) for KNN and Random Forest [31], and LightGBM (version 4.6.0) for gradient-boosted decision trees [32]. All model training and evaluation steps were executed using consistent data partitions and evaluation metrics across runs. Default hyperparameter settings were used for all classifiers, with fixed random seeds where applicable (all fixed at a value of 42), ensuring reproducibility and consistency across experimental runs.

To ensure full reproducibility, all random number generators involved in data partitioning, metaheuristic initialization, and model training were explicitly controlled. The complete experimental pipeline was executed under identical computational settings across all runs.

This implementation strategy ensures transparency, reproducibility, and methodological robustness, facilitating reliable comparison of feature selection outcomes and predictive performance across metaheuristic algorithms and classification models.

## 4. Results

### 4.1. Baseline Results and Reference Benchmarks

This subsection establishes the reference scenarios against which the metaheuristic-driven feature selection framework is evaluated. Three complementary baseline settings are considered: (i) supervised learning models trained on the full feature space without dimensionality reduction, (ii) an interpretable rule-based threshold benchmark reflecting a low-complexity operational strategy, and (iii) traditional feature selection methods based on filtering and embedded mechanisms. Together, these reference configurations provide a structured foundation for assessing the incremental contribution of wrapper-based optimization in terms of predictive performance, dimensionality reduction, and robustness.

#### 4.1.1. Baseline Model Performance Without Feature Selection

This subsection reports the predictive performance obtained using the complete preprocessed feature space under the 80/20 train–test split described in Section 3.8. No dimensionality reduction or feature selection mechanism was applied. These results constitute the reference configuration against which wrapper-based optimization strategies are evaluated.

All classifiers introduced in Section 3.7 were trained on the full set of 76 features and evaluated on the held-out test set. Performance is reported using the minority-class-oriented metrics defined in Section 3.6, with emphasis on precision, recall, and F_1_-score for the non-filing (“Stopped Declaring”) class.

Table 7 summarizes the baseline results. The classifiers exhibit heterogeneous behavior in their capacity to detect minority-class events. LightGBM achieves the strongest minority-class performance, with recall equal to 0.951, precision equal to 1.000, and an F_1_-score of 0.975. These values indicate that nearly all non-filing cases are correctly identified, with no false positive predictions in the test set.

Random Forest attains a minority recall of 0.505 and precision of 0.881, reflecting a more conservative decision boundary that correctly classifies most predicted minority cases but fails to capture approximately half of the true non-filing events. KNN exhibits the lowest minority detection capacity, with recall equal to 0.320 and an F_1_-score of 0.407, indicating substantial difficulty in separating minority cases using distance-based discrimination in the full feature space.

**Figure 3:**
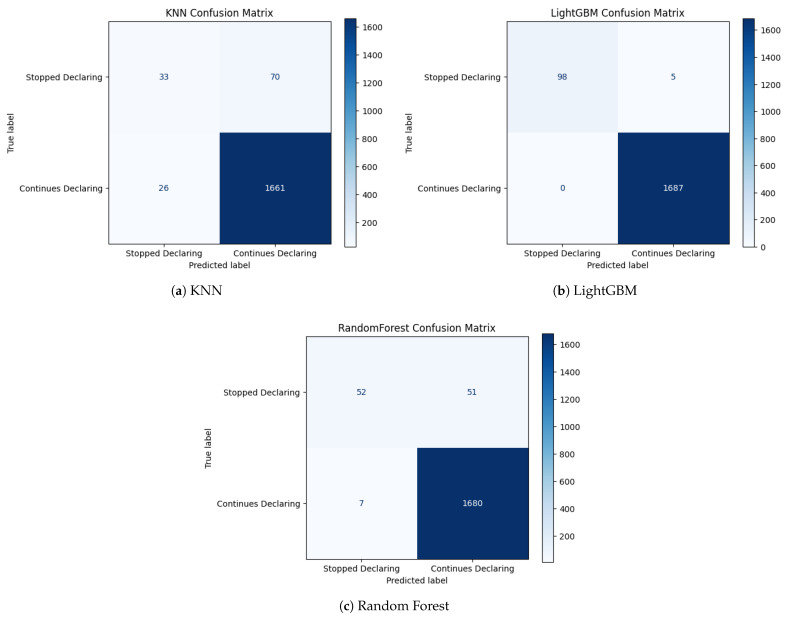
Confusion matrices.

The confusion matrices in Figure 3 provide a class-level view of the error structure. In the test set, the minority class comprises 103 observations, whereas the majority class contains 1687 instances.

For KNN, 70 out of 103 minority cases are misclassified as majority (false negatives), yielding a limited detection rate. Random Forest reduces the number of false negatives to 51 but still fails to identify nearly half of the minority events. In contrast, LightGBM produces only 5 false negatives and no false positives, correctly identifying 98 of 103 non-filing cases while preserving near-perfect majority classification.

Across all models, majority-class performance remains consistently high, with recall values between 0.985 and 1.000. These results confirm that the classification difficulty is concentrated in minority-class detection rather than overall discrimination.

Importantly, the baseline configuration does not exhibit uniformly poor predictive behavior. Instead, performance differences are primarily driven by the inductive bias of each classifier. This baseline therefore establishes a calibrated reference point for subsequent optimization experiments, where feature selection is evaluated not as a corrective measure for global failure, but as a structured mechanism to examine whether minority recall, dimensionality reduction, and stability can be jointly improved.

Table 8 reports the average training and inference times (in seconds) for each baseline classifier using the full 76-feature representation. Training was conducted on 7158 instances, while inference was measured on the test partition of 1790 instances.

All models require only a few seconds to complete the training phase, with KNN exhibiting the lowest training time (1.103 s), followed by LightGBM (3.481 s) and Random Forest (4.380 s). Given that the framework operates on historical administrative data and retraining is performed periodically rather than in real time, these training costs are compatible with institutional batch-processing schedules.

Inference latency is substantially lower, remaining below 0.1 s across all classifiers. Considering that the average number of annual records processed by the institution is approximately 746 cases, these inference times indicate that risk prediction can be executed within negligible computational time under routine operational conditions.

#### 4.1.2. Rule-Based Threshold Baseline Comparison

To establish a fully interpretable operational reference, a rule-based classification strategy was constructed using two institutional variables: deud_monto (total loan amount) and conteo_cuota (number of effective loan installments). Borrowers exceeding the fourth quartile of both variables were classified as potential discontinuers. This deterministic heuristic serves as a low-complexity benchmark and does not involve multivariate learning or parameter estimation.

**Figure 4:**
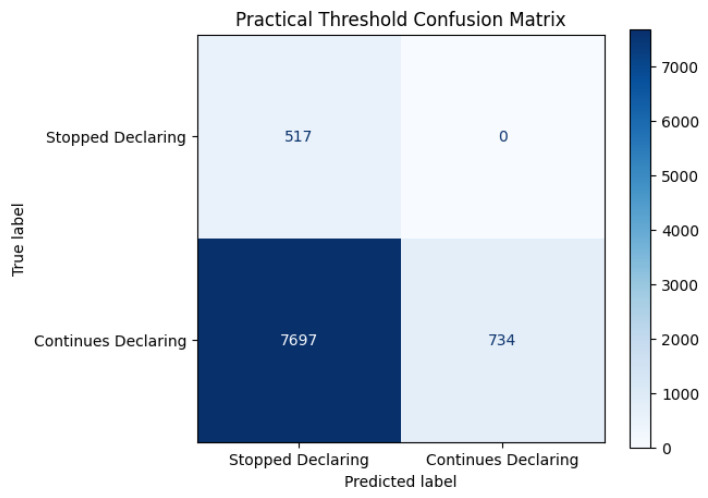
Confusion matrix for the rule-based threshold baseline.

The performance of this strategy, evaluated on the full dataset, is summarized in Table 9, and the corresponding confusion matrix is presented in Figure 4. The rule-based approach attains perfect minority-class recall (1.000), correctly identifying all 517 observed non-filing cases. However, this exhaustive detection is accompanied by a large number of false positives (7697), resulting in a minority precision of 0.063 and an F_1_-score of 0.118.

The majority-class performance further illustrates the imbalance induced by this heuristic. Only 734 out of 8431 compliant borrowers are correctly classified, yielding a majority recall of 0.087. Although majority precision equals 1.000 by construction, this reflects the fact that nearly all predicted majority cases are true negatives, while the vast majority of compliant borrowers are incorrectly flagged as high risk.

This error structure indicates that the rule-based strategy operates as an exhaustive screening mechanism rather than as a discriminative classifier. While operationally simple and fully transparent, the quartile-based threshold fails to capture the multivariate dependencies underlying declaration behavior. The resulting false positive burden would generate substantial monitoring overhead in a practical institutional setting.

When contrasted with the supervised learning baselines reported in Section 4.1.1, even non-optimized multivariate models achieve substantially more balanced precision–recall trade-offs under comparable evaluation conditions. The rule-based benchmark therefore provides a lower bound reference in terms of discriminatory capacity, against which data-driven modeling strategies can be contextualized.

#### 4.1.3. Traditional Future Selection Baselines

To establish a structured benchmark for the subsequent wrapper-based optimization experiments, two conventional dimensionality reduction strategies were applied to the full 76-feature space: Mutual Information (filter method) and L1-based regularization (embedded method). The objective is to quantify how established feature selection mechanisms affect minority-class detection, feature compactness, and computational cost relative to the no-selection baseline presented in Section 4.1.1.

For Mutual Information filtering, variables with zero mutual information with respect to the target label were removed. For L1 regularization, features with coefficients shrunk to zero during model fitting were discarded. These procedures reduced the feature space from 76 variables to 44 (Mutual Information) and 64–65 variables (L1 regularization), depending on the classifier.

Table 10, Table 11 and Table 12 summarize minority-class recall, number of retained features, and total runtime for each classifier.

The effect of traditional feature selection is heterogeneous across models. For KNN, Mutual Information filtering increases minority recall from 0.320 to 0.408 while reducing dimensionality to 44 features. L1 regularization maintains recall at 0.320, indicating limited sensitivity of the distance-based classifier to coefficient-driven pruning. For Random Forest, Mutual Information increases minority recall from 0.505 to 0.592, whereas L1 regularization produces a modest increase to 0.524. Although improvements are observable, they remain moderate in magnitude. For LightGBM, Mutual Information filtering reduces minority recall from 0.951 to 0.728, suggesting that features with low marginal relevance may still contribute to tree-based interaction structures. L1 regularization preserves baseline recall (0.951) while reducing dimensionality to 65 features.

In terms of computational cost, Mutual Information filtering introduces minimal additional overhead relative to baseline training, whereas L1 regularization requires substantially longer runtimes due to coefficient estimation during model fitting.

Overall, traditional feature selection methods produce model-dependent and quantitatively limited effects on minority-class recall. While dimensionality reduction is achieved, no uniform improvement pattern emerges across classifiers. These results provide a controlled reference against which the behavior of metaheuristic-based feature selection can be evaluated.

### 4.2. Predictive Performance After Optimization

This subsection evaluates predictive performance after wrapper-based metaheuristic feature selection under the experimental protocol described in Section 3.9. For each classifier–optimization configuration, performance metrics are computed over repeated stochastic executions in order to account for variability inherent to population-based search procedures. Minority- and majority-class metrics are reported to assess discrimination capacity and class balance under reduced feature representations. All reported averages and standard deviations are computed over 31 independent optimization runs per configuration.

#### 4.2.1. Minority Class

Table 13, Table 14 and Table 15 summarize minority-class predictive performance after metaheuristic-driven feature selection across classifiers and optimization strategies. Minority-class metrics are emphasized in this stage, as they directly reflect the models’ ability to identify income declaration abandonment events, which constitute the primary operational risk in post-declarative institutional settings.

The results indicate a differentiated impact of metaheuristic-driven feature selection across classifiers.

For LightGBM, minority-class performance remains consistently high across all optimization strategies. Average recall values range between 0.935 and 0.943, with low standard deviations (≤0.015). F_1_-scores exceed 0.95 under all optimization schemes, indicating stable discrimination capacity across repeated runs. These results indicate that feature subset optimization largely preserves the strong predictive behavior of gradient boosting while maintaining low variability across runs.

Notably, average minority recall for LightGBM under optimization does not exceed the baseline configuration, indicating that wrapper-based feature reduction does not uniformly improve already well-performing ensemble models.

Random Forest exhibits more heterogeneous behavior. Under PSO, average minority recall reaches 0.955, whereas WOA leads to lower average performance (0.699) and higher dispersion. This sensitivity indicates that ensemble tree methods benefit from structured subset exploration, but outcomes remain dependent on the search dynamics of the metaheuristic employed.

For KNN, the effect of optimization is highly strategy-dependent. GWO produces a marked increase in average minority recall (0.849), whereas PSO and WOA yield more moderate performance levels with higher variability. The observed dispersion confirms that distance-based classifiers remain sensitive to feature subset composition, even under guided search.

#### 4.2.2. Majority Class

Majority-class performance metrics obtained after metaheuristic-driven optimization are reported in Table 16, Table 17 and Table 18.

Table 16, Table 17 and Table 18 report the corresponding results for the majority class.

As expected, performance on the majority class remains consistently high across all classifiers and optimization strategies, with recall, precision, and F_1_-score values close to one and low variability. These results indicate that improvements in minority-class detection are not accompanied by systematic degradation in majority-class performance, preserving overall classification balance under wrapper-based optimization.

Taken together, the metric-based analysis shows that metaheuristic-driven feature selection affects predictive behavior in a classifier-dependent manner. Ensemble-based learners retain high and stable discrimination capacity across optimization strategies, whereas the distance-based model exhibits greater sensitivity to feature subset composition. These findings indicate that the effect of wrapper-based search depends on the interaction between the optimization strategy and the inductive bias of each classifier, rather than producing uniform gains across learning paradigms.

From a cross-classifier perspective, the optimization strategies exhibit different stability profiles. GWO maintains comparatively high minority recall across all classifiers without substantial performance deterioration in any configuration, whereas PSO and WOA achieve peak performance in specific classifier configurations but display larger performance disparities across learners. When jointly considering recall magnitude and dispersion, GWO exhibits comparatively homogeneous behavior across models.

### 4.3. Confusion Matrix Analysis

**Figure 5:**
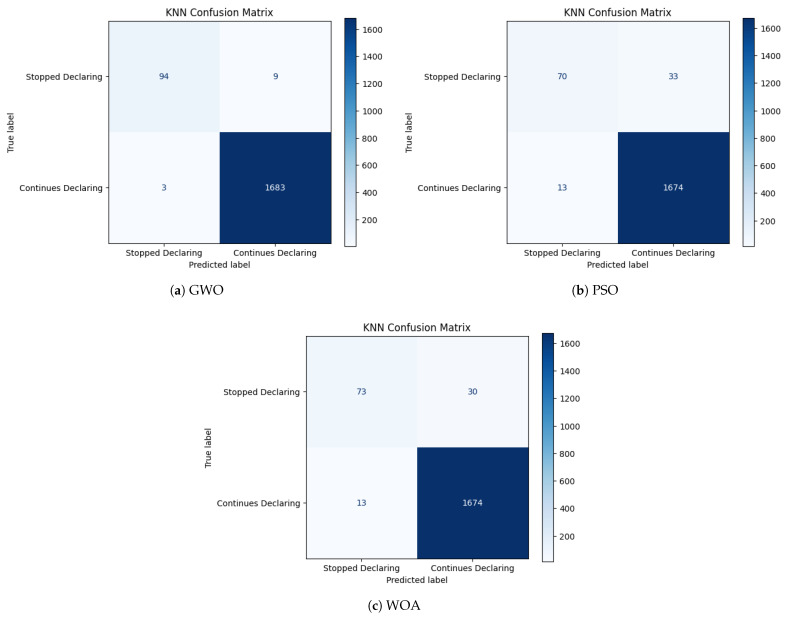
Confusion Matrix KNN.

**Figure 6:**
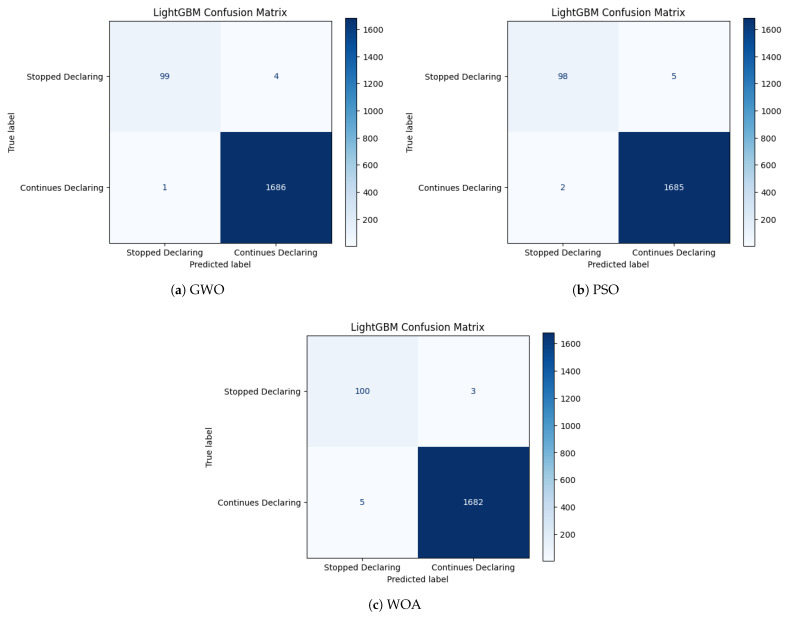
Confusion Matrix LightGBM.

**Figure 7:**
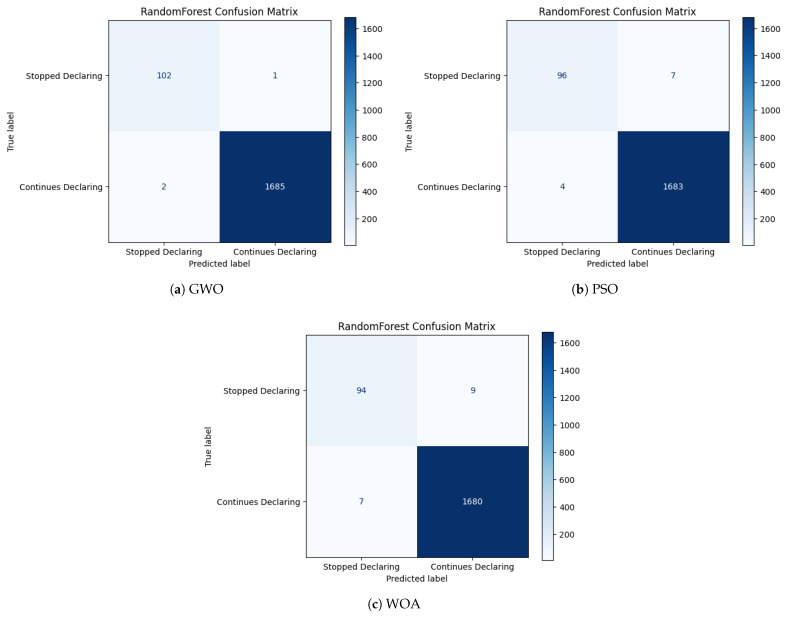
Confusion Matrix Random Forest.

The confusion matrices presented in Figure 5, Figure 6 and Figure 7 provide a complementary, class-level perspective on the predictive behavior of the optimized models analyzed. While aggregate metrics summarize overall performance, confusion matrices enable a direct inspection of error types, particularly false negatives and false positives, which are critical in the context of declaration abandonment prediction. The confusion matrices correspond to the best-performing run obtained under each classifier–optimization configuration.

Figure 5 illustrates the confusion matrices obtained for the KNN classifier under the three metaheuristic optimization strategies. Across configurations, KNN exhibits a systematic tendency to misclassify a non-negligible fraction of minority-class instances as majority-class outcomes, as reflected by the persistent presence of false negatives in all scenarios. This pattern is particularly pronounced under PSO and WOA, where the volume of false negatives visibly increases relative to GWO. The resulting error distribution suggests that distance-based decision boundaries remain highly sensitive to the selected feature subsets, leading to variability in minority-class separation across optimization strategies.

In contrast, the confusion matrices for LightGBM, shown in Figure 6, reveal a markedly more balanced error structure. Across all optimization strategies, false negatives remain scarce and false positives minimal, resulting in a highly symmetric and stable classification pattern. The near-identical error distributions observed under GWO, PSO, and WOA indicate limited sensitivity to the specific feature subset configuration, indicating limited sensitivity to feature subset variation under the evaluated configurations.

Figure 7 presents the confusion matrices obtained for Random Forest under the three metaheuristic optimization strategies. Random Forest demonstrates a strong capacity to detect minority-class instances, but with a more heterogeneous error structure across optimization strategies. While GWO and PSO configurations maintain a limited number of false negatives, the WOA-based configuration exhibits a visibly higher minority miss rate, indicating greater sensitivity to subset composition. This variability suggests that bagging-based ensembles retain high detection capacity but are less structurally stable than boosting models across optimization strategies.

Overall, the confusion matrix analysis complements the aggregate metrics by explicitly characterizing error composition. LightGBM displays consistently low false-negative counts and limited false-positive rates across optimization strategies. Random Forest maintains strong minority detection with moderate variability in error distribution, whereas KNN exhibits comparatively higher false-negative rates under certain configurations. From an institutional compliance perspective, false negatives correspond to undetected declaration abandonment events, which may entail higher downstream administrative and fiscal implications than false positives. These observations highlight the importance of examining error structure in addition to aggregate performance metrics when selecting optimized predictive pipelines.

### 4.4. Feature Selection Outcomes

This subsection reports the feature selection outcomes obtained through a frequency-based analysis of the optimized subsets across repeated runs. The heatmaps summarize, for each of the 76 candidate variables (indexed from 0 to 75; see Table A2 for feature definitions), the number of times a feature was selected over the 31 independent executions of the metaheuristic-driven wrapper process. Feature inclusion frequencies were computed from optimized subsets obtained through wrapper-based search performed exclusively on the training partitions and aggregated per classifier–metaheuristic configuration. Darker blue tones indicate higher selection frequency, corresponding to features consistently retained across runs, whereas lighter yellow tones denote features rarely selected. This representation allows assessing subset stability and identifying variables that are recurrently preferred under different optimization strategies and classifiers.

This subsection is primarily descriptive in nature; comparative performance implications are examined in Section 4.5.

**Figure 8:**
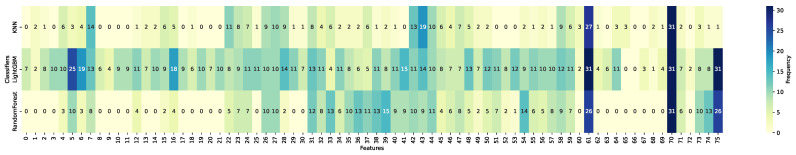
Heatmap features selected by GWO.

**Figure 9:**
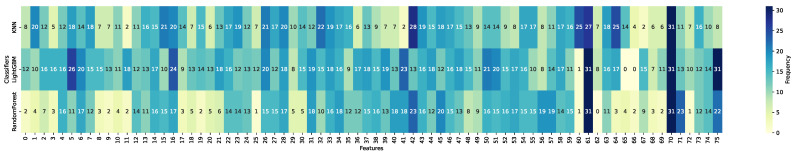
Heatmap features selected by PSO.

**Figure 10:**
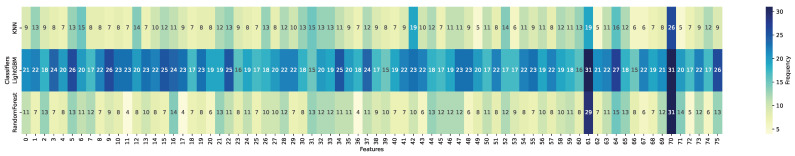
Heatmap features selected by WOA.

Figure 8, Figure 9 and Figure 10 present the selection frequency profiles grouped by metaheuristic, illustrating how GWO, PSO, and WOA distribute feature inclusion frequency across the feature space for KNN, LightGBM, and Random Forest. The visual patterns indicate that selection frequency is highly concentrated on a reduced subset of variables rather than uniformly distributed across the full feature space. This concentration suggests convergence toward a reduced set of recurrent predictors across optimization strategies.

**Figure 11:**
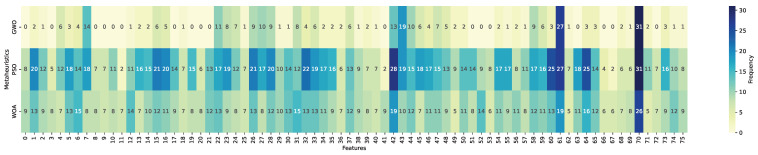
Heatmap Features Used by KNN.

**Figure 12:**
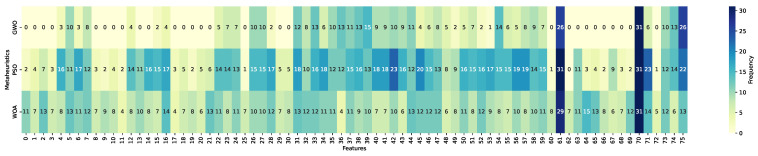
Heatmap features used by Random Forest.

**Figure 13:**
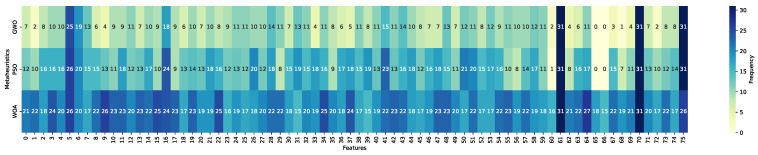
Heatmap features used by LightGBM.

Figure 11, Figure 12 and Figure 13 reorganize the same evidence by classifier, enabling a direct comparison of how each learning model interacts with the metaheuristic-driven feature selection process. From this perspective, ensemble-based models display comparatively homogeneous selection patterns across metaheuristics, whereas KNN exhibits a more heterogeneous distribution of selected features. This reorganization highlights classifier-specific sensitivities to subset composition and optimization dynamics.

A structured interpretation of the heatmap evidence reveals three main structural patterns. First, a compact and recurrent core subset of variables emerges across metaheuristics and classifiers. In particular, num_declaraciones (index 61) and conteo_cuota (index 70) are selected in the majority of the 31 optimization runs, followed by anio_exigibilidad (index 75). These attributes capture complementary behavioral dimensions, including historical declaration engagement, effective repayment activity, and temporal enforcement pressure. Their persistent selection across independent executions indicates recurrent inclusion of a compact subset of predictors across independent runs.

Second, systematic differences emerge across metaheuristics. Particle Swarm Optimization exhibits the highest overall selection intensity, characterized by broader inclusion of features across runs. Whale Optimization Algorithm follows a similar though slightly less expansive pattern. In contrast, Grey Wolf Optimizer produces markedly more compact subsets, indicating comparatively more compact subset construction. These differences are consistent with the distinct search behaviors observed across optimization strategies.

Third, classifier-specific patterns further refine this interpretation. When aggregating cumulative selection frequencies across metaheuristics and runs, LightGBM accumulates a substantially higher total number of feature inclusions (3443) compared to KNN (2101) and Random Forest (2069). Here, a selection denotes the inclusion of a feature in a single optimized subset; thus, these values represent cumulative frequency rather than the number of distinct variables. This pattern indicates that the LightGBM-based wrapper configurations tend to retain larger subsets across runs relative to KNN and Random Forest.

In addition to the core variables shared across classifiers, the gradient boosting model frequently emphasizes features such as deud_e_deuda_1 (index 5) and dein_c_afp_0 (index 16), which are associated with debt magnitude and pension system affiliation, respectively. This pattern suggests that the LightGBM-based configurations tend to retain additional financial and institutional variables beyond the core behavioral predictors.

Overall, the observed patterns indicate that feature selection is non-uniform and exhibits recurrent structure across runs and configurations, rather than reflecting uniform random inclusion across the full feature space. The consistency of frequently selected variables across optimization strategies and classifiers suggests the presence of a structured selection pattern within the evaluated experimental setting.

To complement the frequency-based heatmap analysis and quantify effective dimensionality, Table 19 reports the average number of features selected per optimization run across metaheuristics and classifiers.

Marked differences in subset compactness are observed across metaheuristics. Grey Wolf Optimizer consistently yields the most compact solutions, selecting on average 15.9 features across classifiers. In contrast, Particle Swarm Optimization and Whale Optimization Algorithm retain substantially larger subsets, with average values exceeding 32 features per run. These results quantitatively reinforce the compactness differences observed in the heatmap visualizations.

From a classifier perspective, LightGBM operates with systematically richer feature representations (37.0 features on average), compared to approximately 22 features for both KNN and Random Forest. This pattern is consistent with the ability of gradient boosting ensembles to integrate a larger number of predictors without severe performance degradation.

Beyond structural selection patterns, computational efficiency constitutes an additional dimension of wrapper-based optimization. To complement the compactness analysis, the average offline optimization time required for each metaheuristic–classifier configuration is reported in Table 20. These measurements correspond exclusively to the feature selection phase and do not include inference time.

The results indicate that the classifier embedded within the wrapper is the primary determinant of computational cost. Configurations based on KNN exhibit the lowest optimization times (approximately 475–617 s on average), whereas Random Forest configurations require substantially longer execution times (approximately 2000–2255 s). LightGBM-based wrappers occupy an intermediate range.

Across classifiers, GWO consistently yields the lowest or near-lowest average optimization times for LightGBM and Random Forest, while also maintaining relatively low dispersion in execution time. PSO presents intermediate computational cost, and WOA exhibits higher variability in certain configurations, particularly under Random Forest.

When jointly considering minority-class recall (Table 21), subset compactness (Table 19), and optimization time, GWO represents a computationally efficient wrapper strategy that balances predictive performance and dimensional reduction within the evaluated experimental setting. Importantly, all reported optimization costs are confined to the offline feature selection stage. Once the subset is determined, operational deployment requires only standard classifier inference over the selected variables, thereby separating computationally intensive search from routine decision-support execution.

### 4.5. Comparative Perspective Against Traditional Feature Selection

The objective of this subsection is not to compare metaheuristic algorithms among themselves, but to contrast wrapper-based optimization against traditional feature selection families under a consistent experimental setting. For this purpose, the Grey Wolf Optimizer (GWO) is used as an illustrative wrapper configuration, motivated by its comparatively compact and more stable subset behavior observed in the previous analysis.

When contrasted with the traditional feature selection strategies reported in Table 10, Table 11 and Table 12, the comparative effects of wrapper-based optimization can be assessed under identical data partitions and evaluation procedures. Table 21 summarizes minority-class recall, variation relative to the baseline configuration, and the resulting subset sizes for each classifier.

For KNN, traditional methods yield limited improvements relative to the baseline configuration. Mutual Information increases minority recall from 0.320 to 0.408 (+0.088), while L1 regularization produces no measurable change. In contrast, wrapper-based optimization under GWO increases recall to 0.849 while reducing the feature space from 76 variables to fewer than 10 on average. This pattern indicates that, for a distance-based classifier, recall gains are accompanied by substantial dimensionality reduction.

For Random Forest, Mutual Information filtering improves recall from 0.505 to 0.592 (+0.087), and L1 regularization yields a marginal increase to 0.524 (+0.019). Under GWO-based optimization, minority recall reaches 0.932 (+0.427) while reducing the subset to approximately 14 variables. In this setting, wrapper-based optimization yields both higher minority-class recall and more compact representations.

For LightGBM, baseline minority recall is already high (0.951). Mutual Information filtering leads to a marked degradation (−0.223), and L1 regularization preserves baseline performance. Wrapper-based optimization under GWO yields a minor reduction to 0.937 (−0.014) while reducing the feature space to 24 variables. Here, the main effect is dimensionality reduction with limited change in minority-class recall.

Overall, when jointly considering minority recall and subset compactness, wrapper-based optimization yields the largest gains for classifiers with weaker baseline minority discrimination (KNN and Random Forest). For a strong ensemble learner such as LightGBM, the empirical benefit is concentrated in dimensionality reduction rather than recall improvement.

#### Trade-Off Analysis Between Subset Size and Minority-Class Recall

**Figure 14:**
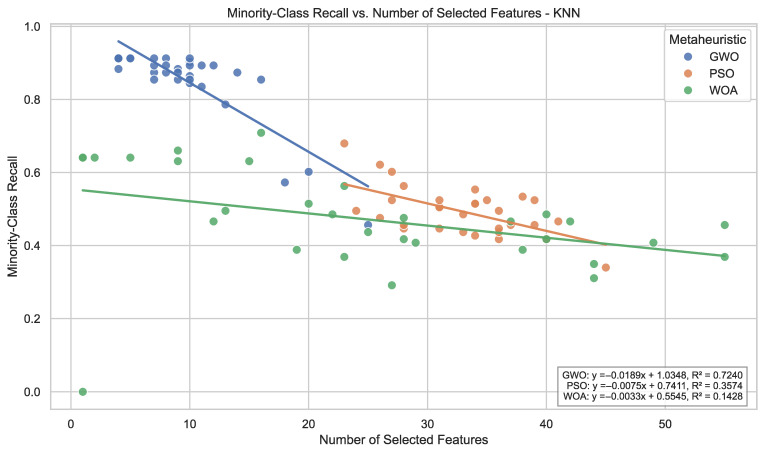
Minority-class recall as a function of the number of selected features for KNN across 31 optimization runs per metaheuristic. Linear trend lines and corresponding $R^{2}$ values are shown to quantify the strength of the association.

**Figure 15:**
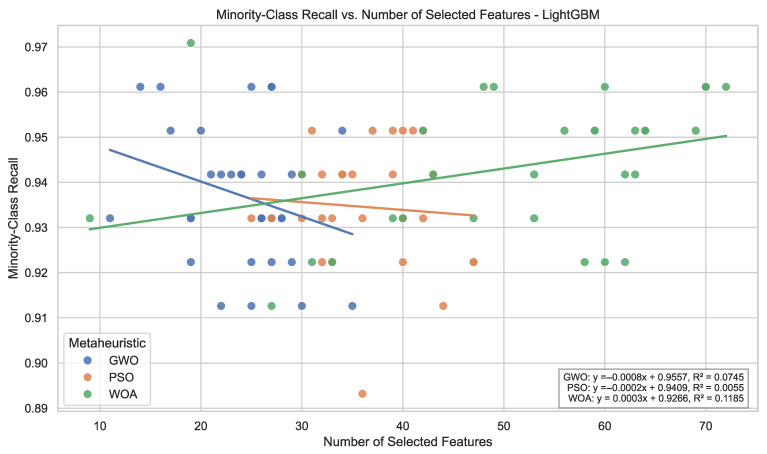
Minority-class recall as a function of the number of selected features for LightGBM across 31 optimization runs per metaheuristic. Linear trend lines and corresponding $R^{2}$ values are shown to quantify the strength of the association.

**Figure 16:**
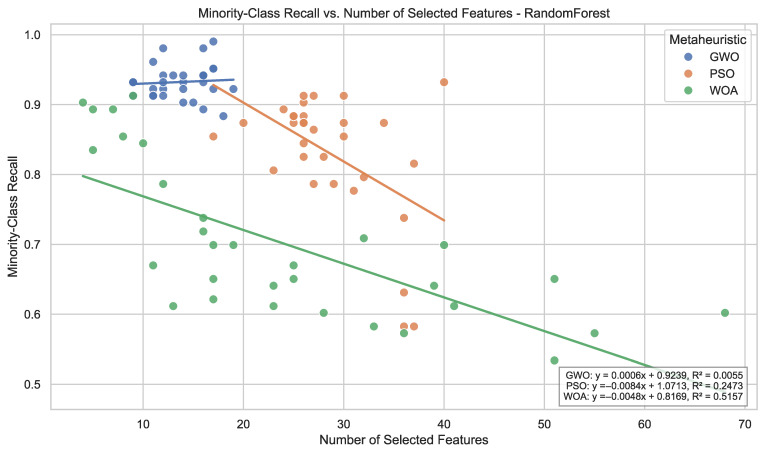
Minority-class recall as a function of the number of selected features for Random Forest across 31 optimization runs per metaheuristic. Linear trend lines and corresponding $R^{2}$ values are shown to quantify the strength of the association.

To further characterize the interaction between subset compactness and predictive behavior, a trade-off analysis evaluated the association between the number of selected features and minority-class recall across the 31 independent optimization runs for each metaheuristic–classifier configuration. Figure 14, Figure 15 and Figure 16 present the corresponding scatter plots together with linear trend estimations.

For LightGBM, the estimated linear relationships are weak across all metaheuristics (all $R^{2}<0.12$). Under GWO, the slope is approximately −0.0008, while PSO and WOA exhibit slopes close to zero. These values indicate that variation in subset size explains little of the variability in minority recall, and no clear linear trend is observed across the evaluated dimensional range. This pattern is consistent with substantial feature compression being achievable without a predictable linear loss in minority detection capacity.

For Random Forest, a moderate negative association is observed under PSO and WOA (with $R^{2}$ reaching approximately 0.52 under WOA), indicating that larger subsets tend to be associated with lower minority recall in those configurations. In contrast, the GWO configuration exhibits minimal linear association, suggesting that the subset-size effect is configuration-dependent.

The strongest dimensionality effect is observed for KNN. Under GWO, minority recall exhibits a pronounced negative slope (−0.0189) with a high coefficient of determination ($R^{2}=0.724$). Similar though weaker trends are observed for PSO and WOA. These results indicate that a substantial portion of recall variability in KNN is associated with dimensionality changes, consistent with the sensitivity of distance-based classifiers to high-dimensional feature spaces.

Taken together, the trade-off analysis shows that the relationship between subset size and minority detection is classifier-dependent rather than uniform. For ensemble-based learners, dimensionality reduction primarily yields structural compression with limited evidence of systematic linear recall degradation. For distance-based classifiers, dimensional compression is associated with improved minority recall.

This analysis complements Table 21 by quantifying the linear association between subset compactness and minority-class recall under repeated experimental conditions.

### 4.6. Robustness and Stability Analysis

This subsection evaluates predictive robustness by analyzing variability in classification-level metrics (recall, precision, and F1-score) across independent optimization runs. Rather than focusing on best-case outcomes, the analysis emphasizes average values and dispersion measures reported in Table 13, Table 14 and Table 15, which provide direct evidence of the consistency of predictive behavior under repeated experimental conditions.

Ensemble-based classifiers exhibit markedly stable minority-class performance after optimization. LightGBM, in particular, maintains high average recall values for the minority class across all metaheuristics (0.937 under GWO, 0.935 under PSO, and 0.943 under WOA), with consistently low standard deviations ranging between 0.013 and 0.015. A similar stability pattern is observed for minority-class F1-scores, whose dispersion remains at or below 0.011 across optimization strategies. These low variability levels indicate that the predictive behavior induced by the metaheuristic selection process remains consistent across runs and largely independent of the specific optimization strategy employed.

Random Forest also demonstrates robust average minority-class performance, although with moderately higher variability across metaheuristics. While GWO- and PSO-based configurations preserve relatively low dispersion (standard deviations below 0.03 for recall), the WOA-based configuration exhibits a noticeable increase in variability (recall standard deviation of 0.111), accompanied by a reduction in average recall. This behavior suggests that Random Forest is more sensitive to variations in selected feature subsets under certain search dynamics.

In contrast, KNN shows substantially higher instability across runs and optimization strategies. Minority-class recall variability is pronounced, with standard deviation values reaching 0.107 under GWO and 0.143 under WOA, alongside sharp reductions in average recall under PSO (0.493) and WOA (0.472). A similar pattern is observed for minority-class F1-scores, confirming that distance-based classifiers are particularly sensitive to fluctuations in feature subset composition in high-dimensional and imbalanced institutional datasets.

Performance stability for the majority class further reinforces these observations. As reported in Table 16 and Table 17, recall, precision, and F1-score values for the majority class remain consistently close to one across all classifiers and optimization strategies, with minimal dispersion. This confirms that improvements in minority-class detection are not achieved at the expense of degraded majority-class performance.

Overall, the robustness analysis indicates that metaheuristic-based feature selection does not inherently induce high performance volatility under the evaluated experimental protocol. Stability differences across classifier families are evident, with ensemble-based methods—particularly LightGBM—exhibiting the most consistent minority-class behavior across independent optimization runs. In contrast, distance-based classifiers display higher sensitivity to subset variation, highlighting that robustness outcomes depend on the interaction between learner architecture and search dynamics rather than on the optimization procedure alone.

### 4.7. Statistical Validation and Algorithm Comparison

This section evaluates whether the observed performance differences among metaheuristic-based feature selection strategies and classifier combinations (i.e., optimized pipelines) are statistically significant, beyond random variation induced by stochastic optimization and data partitioning. The analysis focuses on validating comparative performance across optimized pipelines, rather than on individual metric values, by jointly considering feature selection strategies and predictive models. To this end, non-parametric statistical tests are employed to assess global differences, pairwise comparisons, and consistency of dominance patterns across repeated experimental runs.

This choice is motivated by the absence of normality in performance distributions and by the repeated-measures nature of the experimental design, where multiple algorithms are evaluated across the same datasets, classifiers, and performance metrics. Statistical comparisons are conducted using the primary performance metric defined in Section 3.6, ensuring consistency between optimization objectives, descriptive evaluation, and inferential analysis.

The statistical analysis follows a three-stage comparative procedure commonly recommended for algorithm comparison in machine learning and optimization research [33,34,35]. First, the Friedman test is applied to determine whether there are overall statistically significant differences among the evaluated algorithms across repeated experimental runs. The Friedman test evaluates the null hypothesis that all algorithms exhibit equivalent performance rankings. A significance level of α=0.05 is adopted throughout the analysis.

When the Friedman test indicates a statistically significant global difference (i.e., p<0.05), a Nemenyi post hoc test is subsequently applied to identify pairs of algorithms whose performance differences are statistically significant. This post hoc analysis accounts for multiple comparisons and enables pairwise differentiation while controlling the family-wise error rate.

Finally, for algorithm pairs exhibiting statistically significant differences under the Nemenyi test, the Wilcoxon signed-rank test is employed to determine the directionality of the observed differences. The Wilcoxon test is applied in a paired manner, as all algorithms are evaluated under identical experimental conditions and random seeds. This test allows identifying which algorithm consistently outperforms another in terms of the selected performance metric.

All statistical tests are conducted using the scipy.stats module in Python. The Wilcoxon signed-rank test is configured with a significance level of α=0.05 and an alternative hypothesis consistent with the optimization objective, enabling the identification of statistically superior feature selection strategies. This statistical validation framework complements the descriptive performance analysis and provides strong inferential support.

By applying the Friedman test to all the experiments performed, considering the objective function (OF) defined in Equation (3), we obtained a *p*-value of $5.30\times 10^{-43}$. Since we obtained a *p*-value <0.05, we can conclude that there is an overall statistical difference and we can apply the Neminyi post hoc test to determine the pairs with significant differences.

Table 22 shows the *p*-values obtained after applying the post hoc test. As can be seen, we have 22 pairs with a significant difference (*p*-value <0.05), which will be reviewed using the Wilcoxon signed-rank test.

To apply the Wilcoxon signed-rank test, we must define the comparison assumptions. These assumptions are constructed assuming the objective function is a minimization function and are defined as follows:

(8) $$\begin{array}{c} H_{0}=\mathrm{Feature}{\mathrm{Selector}}_{1}\geq \mathrm{Feature}{\mathrm{Selector}}_{2}\\ H_{1}=\mathrm{Feature}{\mathrm{Selector}}_{1}<\mathrm{Feature}{\mathrm{Selector}}_{2} \end{array}$$

If the test yields a *p*-value <0.05, we can assume that $\mathrm{Feature}{\mathrm{Selector}}_{1}$ performs worse than $\mathrm{Feature}{\mathrm{Selector}}_{2}$; therefore, $H_{0}$ is rejected. In other words, we can say that, statistically, the fitness scores obtained with $\mathrm{Feature}{\mathrm{Selector}}_{2}$ are lower than those obtained with $\mathrm{Feature}{\mathrm{Selector}}_{1}$; therefore, $\mathrm{Feature}{\mathrm{Selector}}_{2}$ is better than $\mathrm{Feature}{\mathrm{Selector}}_{1}$.

Table 23 summarizes the algorithms’ wins, based on the p-values obtained from the statistical test. First, we note that the LGBM classifier achieved the best performance across all three metaheuristics (5 wins among the other algorithms). Second, we highlight the GWO metaheuristic, which achieved 10 wins (2 with the KNN classifier, 5 with the LGBM classifier, and 3 with the RF classifier), making it the best-performing metaheuristic.

Table 24 shows the details of the statistical tests between the pairs with significant differences detected in Table 22. Observing the table, we can highlight the following:

- Dominance of LGBM-based Models: The results consistently demonstrate that the Light Gradient Boosting Machine (LGBM) classifier, when coupled with any of the evaluated metaheuristics (GWO, PSO, or WOA), significantly outperforms configurations based on Random Forest (RF) and K-Nearest Neighbors (KNN) in fitness socres. This suggests that LGBM’s boosting architecture is highly effective for the feature subsets selected by these algorithms.
- hlSuperiority of Grey Wolf Optimizer (GWO): In direct comparisons involving the same classifier, GWO-based feature selection (specifically GWO_KNN) demonstrated a statistically superior performance over its PSO and WOA counterparts. This identifies GWO as a robust search agent for exploring the feature space in this particular problem domain.
- Comparative Performance Hierarchy: The Wilcoxon signed-rank test results establish a clear performance hierarchy where LGBM > RF > KNN across all metaheuristic frameworks. Furthermore, PSO_LGBM and WOA_LGBM showed no performance degradation compared to traditional pairings, confirming the scalability of these hybrid metaheuristic-classifier approaches.

**Table 24:** Wilcoxon signed-rank test for pairs with significant difference.

Comparison	*p*-Value	Conclusion
GWO_KNN v/s PSO_KNN	$3.26\times 10^{-09}$	GWO_KNN is better than PSO_KNN
GWO_KNN v/s WOA_KNN	$4.66\times 10^{-10}$	GWO_KNN is better than WOA_KNN
GWO_LGBM v/s GWO_KNN	$9.31\times 10^{-10}$	GWO_LGBM is better than GWO_KNN
GWO_LGBM v/s PSO_KNN	$4.66\times 10^{-10}$	GWO_LGBM is better than PSO_KNN
GWO_LGBM v/s PSO_RF	$4.66\times 10^{-10}$	GWO_LGBM is better than PSO_RF
GWO_LGBM v/s WOA_KNN	$4.66\times 10^{-10}$	GWO_LGBM is better than WOA_KNN
GWO_LGBM v/s WOA_RF	$4.66\times 10^{-10}$	GWO_LGBM is better than WOA_RF
GWO_RF v/s PSO_KNN	$4.66\times 10^{-10}$	GWO_RF is better than PSO_KNN
GWO_RF v/s WOA_KNN	$4.66\times 10^{-10}$	GWO_RF is better than WOA_KNN
GWO_RF v/s WOA_RF	$4.66\times 10^{-10}$	GWO_RF is better than WOA_RF
PSO_LGBM v/s GWO_KNN	$4.66\times 10^{-10}$	PSO_LGBM is better than GWO_KNN
PSO_LGBM v/s PSO_KNN	$4.66\times 10^{-10}$	PSO_LGBM is better than PSO_KNN
PSO_LGBM v/s PSO_RF	$4.66\times 10^{-09}$	PSO_LGBM is better than PSO_RF
PSO_LGBM v/s WOA_KNN	$4.66\times 10^{-10}$	PSO_LGBM is better than WOA_KNN
PSO_LGBM v/s WOA_RF	$4.66\times 10^{-10}$	PSO_LGBM is better than WOA_RF
PSO_RF v/s PSO_KNN	$9.31\times 10^{-10}$	PSO_RF is better than PSO_KNN
PSO_RF v/s WOA_KNN	$4.66\times 10^{-10}$	PSO_RF is better than WOA_KNN
WOA_LGBM v/s GWO_KNN	$4.66\times 10^{-10}$	WOA_LGBM is better than GWO_KNN
WOA_LGBM v/s PSO_KNN	$4.66\times 10^{-10}$	WOA_LGBM is better than PSO_KNN
WOA_LGBM v/s PSO_RF	$4.66\times 10^{-10}$	WOA_LGBM is better than PSO_RF
WOA_LGBM v/s WOA_KNN	$4.66\times 10^{-10}$	WOA_LGBM is better than WOA_KNN
WOA_LGBM v/s WOA_RF	$4.66\times 10^{-10}$	WOA_LGBM is better than WOA_RF

### 4.8. Analysis of Performance Limitations

Although metaheuristic-driven wrapper feature selection improves minority-class detection in several configurations, the results also reveal relevant limitations and classifier-dependent behaviors that delimit the operational scope of the proposed framework.

First, performance gains are not uniform across classifiers, particularly when the baseline model already operates near a performance ceiling. LightGBM exhibits strong minority-class recall without feature selection (Table 7). After optimization, its average minority recall remains within a narrow range (0.935–0.943 across metaheuristics; Table 13), indicating that wrapper optimization does not improve minority-class recall relative to the baseline, while enabling dimensionality reduction. This pattern is consistent with a performance plateau, where boosting-based ensembles appear to exploit the original feature space efficiently under the evaluated conditions.

Second, robustness differs markedly across classifier families. LightGBM maintains consistently low dispersion across stochastic runs (minority class recall standard deviations between 0.013 and 0.015), whereas KNN exhibits substantially higher variability, reaching a minority class recall standard deviation of 0.143 under WOA. Random Forest also shows increased instability in specific configurations, particularly under WOA (minority class recall recall standard deviation of 0.111). These differences indicate that performance stability varies across classifier families, suggesting that inductive bias interacts with the selected feature subsets.

Third, certain metaheuristic–classifier combinations remain structurally limited despite optimization. For example, KNN combined with PSO and WOA achieves average minority recall values below 0.50, even after wrapper-based feature selection. This demonstrates that feature subset search alone cannot fully compensate for the limitations observed in distance-based classifiers under high-dimensional and imbalanced conditions.

Fourth, subset dimensionality does not translate monotonically into improved minority detection. As shown in Table 19, PSO and WOA frequently retain substantially larger feature subsets than GWO, yet these richer representations do not consistently yield higher average minority class recall. In particular, WOA coupled with LightGBM selects substantially larger subsets (50.8 features on average) compared to the more compact GWO configurations (24.0 features on average), yet yields only a marginal increase in average minority-class recall (0.943 vs. 0.937). This limited performance gain relative to the substantial increase in dimensionality suggests that expanding the feature subset does not lead to proportional improvements in minority detection when ensemble learners already achieve strong baseline performance.

Overall, these findings clarify that the proposed framework does not guarantee uniform improvements across all classifier–metaheuristic combinations. Its effectiveness depends on the interaction between stochastic search dynamics, subset compactness, and the inductive bias of the classifier. Explicitly reporting these performance boundaries strengthens methodological transparency and provides a realistic basis for selecting operationally suitable pipelines in institutional compliance modeling contexts.

## 5. Discussion

### 5.1. Comparative Perspective Across Predictive Settings

The empirical results reveal a consistent differentiation between baseline predictive configurations operating on the full feature space and optimized pipelines incorporating metaheuristic-driven feature selection. While baseline models already capture relevant signals associated with income declaration behavior, their performance may be affected by feature redundancy, noise, and class imbalance. The incorporation of wrapper-based optimization modifies how the available information is structured and exploited, primarily through controlled dimensionality reduction and imbalance-aware objective functions.

From a data standpoint, the post-compliance prediction setting examined in this study incorporates longitudinal declaration trajectories, repayment dynamics, and temporal enforceability patterns. This enrichment increases both dimensionality and heterogeneity of the feature space. However, the baseline evaluation shows that additional variables alone do not guarantee improved minority-class detection and that minority-class performance can remain heterogeneous across learning paradigms when the full feature space is retained.

The integration of metaheuristic-driven feature selection addresses this issue by explicitly optimizing subset composition under imbalance-aware criteria. Because baseline and optimized configurations are evaluated under identical data partitions and consistent evaluation protocols, the observed differences are consistent with the effect of the optimization process rather than changes in data availability or evaluation design. The results therefore indicate that feature subset refinement can contribute to controlled dimensionality and improved minority-class behavior in specific classifier–metaheuristic configurations, while stability patterns remain classifier-dependent.

Rather than functioning solely as a mechanism for maximizing isolated performance metrics, the optimization framework operates as a structured filtering process that prioritizes subsets aligned with the selected objective function while limiting redundancy. In this context, metaheuristic-driven feature selection emerges as a methodologically justified enhancement for high-dimensional institutional prediction tasks, particularly when stability and parsimony are considered alongside predictive accuracy.

### 5.2. Interpretability, Parsimony, and Behavioral Coherence

Beyond aggregate predictive performance, the analysis of optimized feature subsets provides structural insight into the behavioral patterns associated with income declaration abandonment. As shown in the frequency-based analysis of Section 4.4, a recurrent subset of variables emerges across metaheuristic algorithms, classifiers, and repeated runs. In particular, features related to declaration continuity, repayment exposure, and temporal enforceability repeatedly exhibit high selection frequency. This cross-configuration recurrence suggests convergence toward a consistent discriminative structure rather than arbitrary subset combinations.

The observed dimensionality reduction further reinforces this interpretation. In several classifier–metaheuristic configurations, optimized pipelines maintain or substantially improve minority-class recall while operating on smaller subsets compared to the original 76-variable feature space. For example, under GWO, average subset sizes decrease to 9.8 features for KNN, 13.7 for Random Forest, and 24.0 for LightGBM. For LightGBM specifically, minority-class recall remains close to the baseline level while dimensionality is reduced, indicating that feature compactness can be achieved without severe degradation in predictive performance. Rather than expanding the representation, the wrapper-based optimization process concentrates predictive signal into reduced subsets, particularly for classifiers with weaker full-feature baselines.

From a computational standpoint, the reduction in subset size is also operationally meaningful. As reported in Table 19, the average number of selected features per run ranges between approximately 15.9 and 33.3 depending on the optimization strategy, representing a substantial contraction of the original feature space. Such dimensionality control reduces feature evaluation requirements at inference time and limits sensitivity to irrelevant variables, while predictive stability remains configuration-dependent across classifiers.

Although metaheuristic search introduces additional algorithmic complexity during the offline optimization phase, the resulting deployed models remain structurally transparent in terms of retained variables. The recurrent selection of administratively interpretable features indicates that bioinspired optimization can be aligned with domain knowledge and governance requirements when guided by a clearly defined objective function. In this setting, parsimony operates as an interpretability mechanism and a practical enabler for institutional integration, while robustness outcomes remain dependent on the interaction between classifier family and search strategy.

### 5.3. Robustness and Methodological Implications

The robustness analysis reported in Section 4.6 indicates that performance stability varies across classifier families and optimization strategies. Ensemble-based classifiers, particularly LightGBM, maintain low dispersion in minority-class recall across metaheuristics, with standard deviations between 0.013 and 0.015, indicating reproducible behavior under stochastic search dynamics. In contrast, stability is configuration-dependent for other learners, as illustrated by Random Forest under WOA (std = 0.111) and KNN under WOA (std = 0.143). These patterns suggest that optimization does not uniformly guarantee stability, but can converge toward structurally consistent predictive behavior for specific classifier–metaheuristic combinations.

Complementarily, the comparative analysis in Table 21 shows that wrapper-based optimization yields substantial improvements in minority-class recall relative to full-feature baselines for selected learners. For KNN, recall increases from 0.284 to 0.849 (+0.565) under GWO, and for Random Forest from 0.471 to 0.932 (+0.461), representing pronounced improvements over the baseline configuration. These gains correspond to average performance across repeated optimization runs rather than isolated best-case realizations, indicating a systematic reshaping of decision boundaries through subset refinement in these cases.

For ensemble-based classifiers, performance changes are more moderate in magnitude and must be interpreted relative to strong baselines. LightGBM achieves a baseline minority-class recall of 0.951 using the full feature space, while optimized recall values range between 0.935 and 0.943 depending on the metaheuristic. In this setting, feature selection does not improve minority-class recall relative to the baseline but preserves competitive performance while operating on substantially reduced feature subsets. The primary contribution of optimization for LightGBM therefore lies in dimensionality control and stable behavior rather than in recall maximization.

Taken together, these findings delineate a practical boundary for optimization-based gains within classical modeling paradigms. When baseline learners already capture most of the discriminative structure present in enriched feature spaces, wrapper-based feature selection primarily regularizes the representation and controls dimensionality. Conversely, for variance-prone or distance-based models, feature selection can substantially reshape decision boundaries and yield pronounced minority-class improvements.

Overall, metaheuristic-driven feature selection emerges as a principled mechanism for enhancing robustness and controlling dimensionality in high-dimensional post-compliance prediction tasks, although its effect on minority-class recall depends on the inductive bias of the underlying classifier. The results further suggest diminishing returns as baseline performance approaches empirical upper bounds under the given data representation, motivating the exploration of more expressive modeling architectures when additional gains are required.

Although the empirical evaluation is grounded in a specific administrative context, the methodological implications extend beyond the analyzed dataset. The combination of imbalance-aware objective functions, repeated stochastic optimization, and wrapper-based subset refinement constitutes a transferable strategy for post-event prediction problems characterized by high dimensionality, temporal structure, and asymmetric error costs. The contribution of this work therefore resides in the demonstrated behavior of optimization-driven feature selection under operational constraints rather than in the universality of individual predictors.

### 5.4. Operational Deployment Considerations

**Figure 17:**
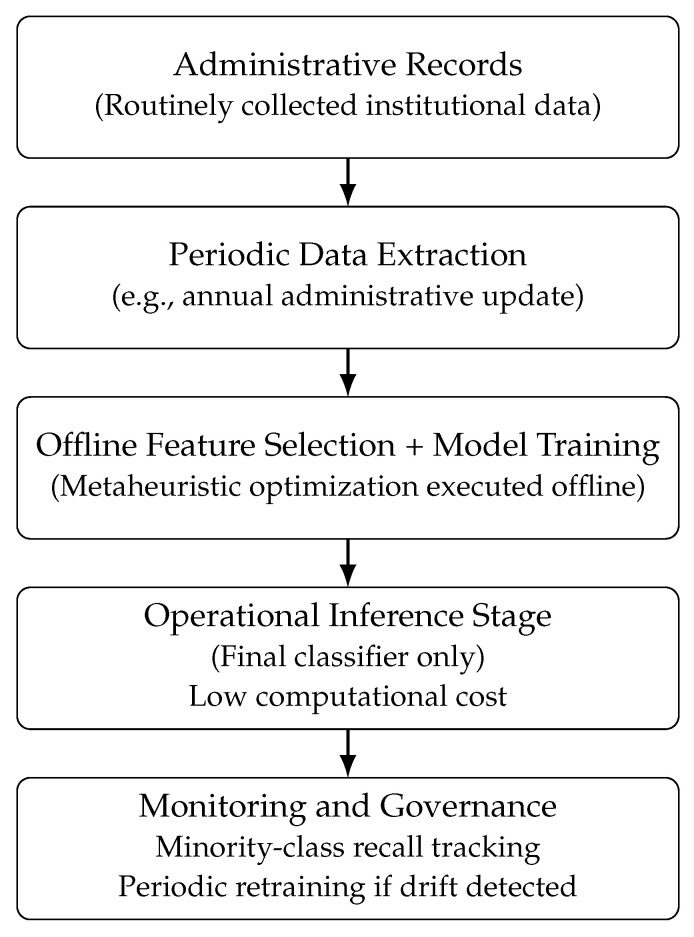
Conceptual operational deployment workflow.

This subsection discusses the operational considerations associated with potential deployment of the proposed framework within institutional administrative workflows. As illustrated in Figure 17, the architecture enforces a strict separation between offline optimization and operational prediction stages.

The considerations presented below are derived from the experimental design and constraints examined in this study. They reflect structural compatibility inferred from the empirical protocol rather than evidence obtained from live production deployment.

#### 5.4.1. Institutional Integration Example: Annual FSCU Operational Cycle

To illustrate the structural compatibility of the proposed framework with an existing institutional decision-support process, this subsection situates the workflow within the documented annual operational cycle of the FSCU at a Chilean university.

Under current FSCU operational regulations [36,37], and consistent with publicly documented institutional procedures at the *Pontificia Universidad Católica de Valparaíso* [38], the administration follows a structured yearly cycle that includes: (i) income declaration submission by beneficiaries (typically completed by the end of May), (ii) institutional validation and database updating during the following months, (iii) compliance monitoring and reporting, and (iv) periodic publication of debt and enforceability records. These stages generate a stable annual snapshot of structured administrative data.

Within this operational context, the predictive framework is structurally compatible with the following sequence:

- **May–June: Cohort Freeze and Data Extraction.** After the declaration deadline, the institutional database contains a complete cohort of updated administrative records. A batch extraction of structured variables can be performed without interfering with transactional systems.
- **June–July: Offline Optimization and Model Training.** Metaheuristic-based feature selection and supervised model training are executed in an isolated analytical environment using the frozen cohort. This phase operates on static data and does not require interaction with live administrative processes.
- **August–September: Batch Risk Scoring.** Once the optimized feature subset and final classifier are validated, inference can be executed as a batch scoring process within the existing compliance-monitoring pipeline.
- **End-of-Year Review: Monitoring and Retraining Decision.** Minority-class recall and related performance indicators can be evaluated against updated administrative records. If statistically significant performance degradation is detected, a new offline optimization cycle can be scheduled.

This calendar-based example demonstrates temporal alignment between the experimental protocol and routine institutional reporting windows. Importantly, the empirical design of this study enforces strict temporal separation between training and prediction, mirroring the batch-based nature of the FSCU cycle. No real-time execution of metaheuristic algorithms is required during operational use, and no modification of transactional database structures is assumed.

The integration scenario described above is derived from documented regulatory procedures and routine administrative reporting cycles. However, the study does not include live production deployment, and therefore does not provide empirical validation of institutional implementation. The analysis instead demonstrates that, under the evaluated experimental conditions, the proposed framework is structurally compatible with periodic administrative workflows.

#### 5.4.2. Operational Impact of Feature Space Reduction

Beyond architectural compatibility, the empirical results provide quantitative evidence regarding the trade-off between predictive performance and feature dimensionality. The original dataset comprised 76 structured variables derived from institutional records and income declaration forms. Under the GWO + LightGBM configuration, optimized subsets with as few as 14–16 variables achieved a minority-class recall of 0.961, compared to a baseline recall of 0.951 obtained using the full feature space.

This corresponds to an absolute improvement of approximately one percentage point in minority recall while reducing dimensionality by 81.6%. From a statistical perspective, the performance gain is moderate. However, the dimensional compression is substantial, indicating that predictive discrimination in this context does not require the full set of administratively available variables.

A considerable portion of the original feature space is derived from annual income declaration forms and associated verification procedures. While this study does not measure administrative processing time or workload, the reduction in required predictors implies that operational deployment would depend on a smaller subset of structured variables during batch risk scoring. In principle, this may reduce the dependency of the predictive pipeline on extensive data extraction and preprocessing routines during annual processing cycles. The magnitude of any resulting administrative efficiency gains, however, would require separate institutional evaluation.

Table 25 presents the most parsimonious high-performing configuration obtained under the GWO + LightGBM setting (Run 5), which achieved a minority-class recall of 0.961 using only 14 variables. Among the top-ranked runs, this configuration illustrates the strongest dimensionality reduction without observable degradation in minority detection performance relative to the full feature baseline.

Because several predictors correspond to one-hot encoded categories derived from structured administrative variables, Table 25 groups the selected features according to their underlying administrative construct and data source.

As shown in Table 25, the retained predictors are primarily longitudinal and administratively stable indicators. Most variables correspond to declaration history, enforceability timing, and installment behavior rather than transient socio-demographic descriptors. This structural concentration suggests that predictive signal is captured predominantly by routinely maintained and institutionally validated variables.

A closer comparison between the optimized subset and the publicly available income declaration form of the FSCU system [38] reveals that several manually declared and annually updated fields are not required by the high-performing configuration. In particular, the selected subset does not rely on detailed monthly income entries, total declared annual income, spousal income information, notarization fields, or other verification-dependent attributes present in the declaration workflow.

Instead, predictive discrimination is predominantly driven by historically aggregated registry variables such as declaration continuity, number of prior declarations, installment behavior, and enforceability timing. These variables are maintained within internal institutional databases and do not require annual revalidation through external documentation processes.

From an operational perspective, this shift implies that the predictive pipeline can rely more heavily on structurally stable, system-generated records rather than on manually submitted declaration components. Although this study does not quantify administrative workload or processing time, the reduced dependence on transient declaration-level variables suggests potential simplification of data extraction routines, lower integration complexity with external entities, and reduced exposure to inconsistencies arising from annual reporting variability.

Overall, the empirical evidence demonstrates that wrapper-based metaheuristic feature selection can preserve minority-class detection performance while substantially reducing feature dimensionality. The operational relevance of this reduction lies in structural simplification of the predictive representation. However, quantitative assessment of administrative cost reduction or workflow acceleration remains outside the scope of this study and would require dedicated institutional evaluation.

#### 5.4.3. Data Availability and Extraction Protocol

The framework operates exclusively on routinely collected administrative records generated within the income-contingent loan management system evaluated in this study. All variables used in the predictive pipeline correspond to structured fields already stored in institutional databases, including declaration history, repayment activity, and temporal enforceability indicators.

Within the scope of the dataset analyzed here, no additional data acquisition procedures were required beyond standard administrative records. Periodic data extraction can be implemented through scheduled queries or batch exports aligned with existing reporting cycles.

Accordingly, under the evaluated data structure, the framework does not depend on external, survey-based, or experimentally generated variables. The extent to which this compatibility generalizes to other institutional contexts would depend on the availability and quality of comparable structured records.

#### 5.4.4. Offline Training and Feature Selection Phase

The metaheuristic-based feature selection process is executed offline and does not require real-time interaction with operational systems. Feature subset optimization and model training are conducted in an isolated analytical environment, separate from transactional databases.

Nature-inspired optimization algorithms such as PSO, GWO, or WOA are not executed during operational inference. Their role is restricted to the offline selection of optimized feature subsets under imbalance-aware objectives. Once a subset is selected and validated, the final predictive model is trained using standard supervised learning procedures.

This architectural separation confines the computationally intensive search process to periodic retraining phases and ensures that optimization routines are not embedded within real-time administrative workflows.

#### 5.4.5. Inference Stage and Computational Cost

After completion of the offline feature selection phase, operational deployment involves only execution of the final trained classifier using the optimized feature subset. No metaheuristic search is performed during inference, and therefore computational cost at deployment corresponds exclusively to standard classifier prediction.

Table 26 reports the average inference time (in seconds) for models trained on feature subsets selected by each metaheuristic. Across all configurations, inference time remains below 0.1 s for the evaluated test partition (1790 instances), with LightGBM exhibiting the lowest latency (approximately 0.002–0.003 s), followed by Random Forest (approximately 0.042 s) and KNN (approximately 0.067–0.075 s).

For comparison, baseline models trained on the full 76-feature space (Table 8) exhibit inference times of similar magnitude. These results indicate that dimensionality reduction does not materially increase deployment latency and that wrapper-based optimization primarily affects offline retraining cost rather than batch inference time.

Accordingly, the computational trade-off introduced by metaheuristic feature selection is concentrated in periodic optimization cycles, while operational risk scoring remains limited to low-latency classifier execution under the evaluated cohort size.

#### 5.4.6. Monitoring and Model Updating Strategy

Operational integration of the proposed framework would necessitate periodic performance monitoring, particularly of minority-class recall, given its role as the primary imbalance-aware evaluation metric in this study. Under a batch-based administrative cycle, predictive performance can be reevaluated on updated annual cohorts using the same validation protocol applied in the experimental design.

Drift detection may involve tracking changes in recall, precision, and class-conditional feature distributions across successive cohorts. While the present study does not implement a formal concept-drift detection mechanism, the evaluation framework used here provides the necessary metrics to support such monitoring if adopted in practice.

Retraining and re-optimization of feature subsets can be scheduled periodically (e.g., annually) or triggered by observed performance degradation relative to predefined institutional thresholds. Because feature selection and model retraining are executed offline, updating the predictive model does not require embedding optimization routines within real-time decision-support systems.

The feasibility of uninterrupted updating is inferred from the architectural separation between offline optimization and batch inference demonstrated in the experimental design. However, operational continuity under institutional deployment would depend on implementation-specific governance and infrastructure constraints that were not evaluated in this study.

#### 5.4.7. Governance, Interpretability, and Auditability

The optimized feature subsets obtained through wrapper-based metaheuristic selection are substantially smaller than the original 76-variable representation and consist primarily of longitudinal administrative indicators. Across optimization runs, retained variables consistently correspond to institutionally defined constructs such as declaration continuity, repayment exposure, and enforceability timing.

From a structural perspective, reduced dimensionality and reliance on administratively defined variables may simplify traceability relative to high-dimensional or representation-learning approaches. However, this study does not formally evaluate governance workflows, audit procedures, or regulatory documentation processes. The interpretability claim is therefore limited to the observable semantic coherence of the selected predictors and their direct correspondence to existing administrative fields.

The empirical results demonstrate that predictive performance can be maintained under substantial dimensional compression without introducing latent representations or opaque feature transformations. This property distinguishes the wrapper-based approach from black-box representation learning methods that may require post hoc explanation techniques.

At the same time, institutional auditability depends on context-specific governance standards, documentation protocols, and compliance requirements that extend beyond the scope of this experimental evaluation. While the architectural separation between offline optimization and batch inference suggests compatibility with periodic risk-scoring cycles, operational validation within a regulated administrative environment would require formal implementation, testing, and approval procedures.

Accordingly, the contribution of this study lies in demonstrating that imbalance-aware predictive performance can be achieved using compact and semantically grounded feature subsets. The implications for governance and audit processes are structurally plausible but were not empirically validated within a live institutional setting.

### 5.5. Theoretical Implications

The empirical results provide structurally grounded insights into the behavior of metaheuristic-driven wrapper feature selection under class imbalance and high-dimensional administrative data. These implications are derived from the experimental evidence reported in Section 4.5 and Section 4.6, and should be interpreted strictly within the evaluated modeling configurations and data conditions.

First, the findings suggest that, under imbalance-aware objective functions, wrapper-based metaheuristic optimization may operate as a stabilization mechanism rather than exclusively as a peak-performance maximizer. As shown in Section 4.6, ensemble-based classifiers such as LightGBM exhibit low dispersion in minority-class recall across independent optimization runs (standard deviations between 0.013 and 0.015), even when selected feature subsets differ. This pattern is consistent with convergence toward regions of the feature space that preserve minority-class discrimination capacity across stochastic realizations. In this setting, optimization appears to reinforce stable predictive representations aligned with recall-oriented objectives, rather than identifying isolated high-performing subsets.

Second, the results reveal a classifier-capacity interaction effect. When baseline learners already capture a large portion of the discriminative structure in the full feature space, as observed for LightGBM (baseline minority recall of 0.951), wrapper-based optimization yields limited recall variation but substantial dimensional compression. In contrast, classifiers with lower baseline discrimination or higher sensitivity to irrelevant features, such as KNN, exhibit pronounced performance gains after feature selection. This differential response suggests that the effect of metaheuristic-driven wrapper optimization is conditioned by the inductive bias and representational robustness of the underlying learner. In some cases, optimization primarily removes redundancy while maintaining recall; in others, it materially reshapes decision boundaries in high-dimensional spaces.

Third, the trade-off analysis between subset size and minority-class recall provides evidence regarding the geometry of the performance landscape explored during wrapper search. For LightGBM, the association between dimensionality and recall is weak across metaheuristics (all $R^{2}<0.12$), indicating a relatively flat response surface along the feature-count axis within the evaluated range. In contrast, KNN exhibits a strong negative association under certain configurations (e.g., $R^{2}=0.724$ under GWO), revealing a steep degradation in recall as dimensionality increases. These heterogeneous patterns imply that wrapper-based optimization does not operate over a uniform search topology; rather, the effective landscape is shaped by classifier architecture and its sensitivity to redundant or irrelevant features.

Taken together, these findings refine the theoretical interpretation of metaheuristic-driven wrapper feature selection in imbalanced applied domains. Rather than functioning solely as a mechanism for maximizing minority-class recall, wrapper optimization can be interpreted as a representation-shaping process whose observable impact depends on objective alignment, learner capacity, and dimensional sensitivity. These conclusions are empirically grounded in the evaluated dataset, imbalance ratio, and classifier families, and do not imply universal properties of wrapper-based optimization. Within the analyzed setting, the theoretical contribution lies in characterizing how search-based subset refinement interacts with learner-induced performance landscapes under imbalance constraints.

## 6. Conclusions

This study examined the behavior of metaheuristic-driven wrapper feature selection as a nature-inspired optimization strategy within a post-compliance income declaration prediction setting. Using real-world administrative data and a repeated stochastic validation framework, the approach was evaluated across multiple classifiers and bio-inspired optimization strategies under realistic class imbalance conditions.

The empirical results show that wrapper-based metaheuristic optimization can materially alter minority-class performance depending on the baseline capacity of the underlying learner. For classifiers with weaker discrimination under the full feature space, such as KNN and certain Random Forest configurations, substantial recall gains are observed relative to baseline models. In contrast, for ensemble-based learners operating near high baseline recall levels (e.g., LightGBM), optimization yields limited changes in minority-class recall but enables pronounced dimensionality reduction. Across configurations, optimized subsets retain on average between approximately 15.9 and 33.3 features per run, compared to the original 76-variable representation. These results indicate that, within the evaluated setting, performance improvements and stability patterns are associated with selective subset refinement rather than expansion of the feature space.

Beyond predictive performance, repeated optimization runs reveal a recurrent subset of administratively meaningful variables related to declaration continuity, repayment exposure, income evolution, and enforceability timing. The consistent reappearance of these constructs across classifiers and optimization strategies supports the structural interpretability of the optimized representations within the analyzed dataset.

Several limitations define the scope of these conclusions.

First, the empirical evaluation is based on a single dataset derived from Chile’s income-contingent student loan system. Although longitudinal and operationally realistic, the transferability of the findings to other compliance domains, such as tax compliance or credit default prediction, remains unverified.

Second, the analysis is restricted to a binary classification setting and does not examine multi-class or multi-risk stratification scenarios.

Third, class imbalance is addressed exclusively through representation-level optimization. The potential interaction between wrapper-based feature selection and data-level imbalance mitigation techniques (e.g., synthetic oversampling) is not explored.

Fourth, the metaheuristic algorithms were executed under fixed population sizes and iteration limits to ensure comparable computational budgets across repeated runs. A dedicated parameter sensitivity analysis was not conducted. Future work may examine how alternative search budgets influence recall, subset compactness, and optimization stability.

Finally, although the architectural design mirrors a batch-based institutional workflow, the study does not include live production deployment or real-time institutional validation.

These limitations delineate several research directions. Extending the empirical evaluation to multiple institutional datasets would allow assessing robustness across administrative contexts. Hybrid approaches integrating wrapper-based feature selection with imbalance-handling strategies could clarify the joint effect of representation refinement and class distribution adjustment. Multi-objective formulations that jointly consider minority recall, subset compactness, and performance dispersion may further refine trade-off analysis in high-stakes compliance prediction settings.

## Figures and Tables

**Table 1:** Cohort selection and filtering process.

Filtering Step/Criterion	Remaining	Removed
Initial dataset (raw debtor administrative records)	68,780	–
Exclude records without a valid loan	44,833	23,947
Restrict to borrowers with at least one observed income declaration	23,170	21,663
Keep borrowers with a well-defined Promissory Note registry	22,629	541
Exclude borrowers with insufficient historical trajectory to define longitudinal features	22,043	586
Exclude out-of-scope or administratively invalid cases	8948	13,095
Final analytic cohort	8948	–

**Table 2:** Descriptive characteristics of the analytic dataset.

Characteristic	Value	Notes
Number of borrowers	8948	Total number of individual borrowers in the cohort.
Declaration discontinuation cases (%)	0.057%	Minority (negative) class proportion.
Declaration compliance cases (%)	0.943%	Majority (positive) class proportion.
Total number of features	52	Before preprocessing and feature selection.
Numerical features	26	Continuous and discrete numerical attributes.
Categorical/Boolean features	24	Encoded categorical and binary attributes.
Time-derived features	2	Aggregated temporal and behavioral indicators.
Observation period (years)	2012–2024	Based on the last observed income declaration.

**Table 3:** Final feature set: variable names, data types, and ranges.

Name	Data Type	Feature Type	Detail
estado_civil	Boolean	Categorical	1 and 2
nacionalidad	Boolean	Categorical	1 and 2
sexo	Boolean	Categorical	1 and 2
fecha_nacimiento	Date	Date	1 January 1900 to 26 March 1991
deud_t_deuda	Integer	Categorical	1 value
deud_e_deuda	Integer	Categorical	7 values
deud_monto	Float	Numerical	1.50 to 1285.76
deud_monto_cuota_fija	Float	Numerical	0.01 to 84.79
deud_fecha_exigibilidad	Date	Date	1 January 2012 to 1 Jannunary 2023
deud_numero_cuotas_fija	Integer	Numerical	0 to 15
paga_t_pagare	Integer	Categorical	1 value
paga_e_pagare	Integer	Categorical	1 value
monto_total_pagare	Float	Numerical	1.37 to 922.22
conteo_pagare	Integer	Numerical	1 to 29
num_declaraciones	Integer	Numerical	1 to 15
dejo_declarar	Boolean	Categorical	0 and 1
anio_ult_declaracion	Integer	Categorical	13 values
dein_c_afp	Integer	Categorical	12 values
dein_estado_civil	Boolean	Categorical	1 and 2
dein_hijo	Boolean	Categorical	0 and 1
dein_c_inst_conyuge_deud	Integer	Categorical	18 values
dein_c_afp_conyuge	Integer	Categorical	10 values
dein_total_deudor_2	Float	Numerical	0 to 4239.44
dein_total_conyuge_2	Float	Numerical	0 to 2487.19
deuda_conyuge	Boolean	Categorical	0 and 1
anio_primera_declaracion	Integer	Categorical	16 values
proporcion_ingreso_cuota	Float	Numerical	−1 to 242.16
prom_proporcion_ingreso_deuda	Float	Numerical	0 to 101.57
std_proporcion_ingreso_deuda	Float	Numerical	0 to 14.92
dif_ingresos_declarados	Float	Numerical	−580.04 to 2582.33
prom_deudor	Float	Numerical	0 to 4239.449
std_deudor	Float	Numerical	0 to 1130.33
prom_conyuge	Float	Numerical	0 to 2469.92
std_conyuge	Float	Numerical	0 to 1785.70
cantidad_cambios_estado_civil	Integer	Numerical	0 to 6
cuot_saldo	Float	Numerical	0 to 84.79
cuot_e_cuota	Integer	Categorical	5 values
avg_valor_cuota	Float	Numerical	0 to 80.36
conteo_cuota	Integer	Numerical	1 to 26
prom_cuota	Float	Numerical	0.28 to 80.19
std_cuota	Float	Numerical	0.12 to 33.09
bole_monto_deuda_prom	Float	Numerical	0.03 to 8.05
bole_count_prom	Float	Numerical	6.4 to 10
conteo_boletin	Integer	Numerical	10 to 366
matr_anio	Integer	Categorical	13 values
matr_c_carrera	Integer	Categorical	74 values
matr_e_matricula	Integer	Categorical	1 value
carr_t_carrera	Integer	Categorical	1 value
matr_c_institucion	Integer	Categorical	1 value
conteo_matr	Integer	Numerical	1 to 31
facultad	Integer	Categorical	9 values
escuela	Integer	Categorical	34 values

**Table 4:** Feature set: detailed descriptions of each variable.

Name	Detail
estado_civil	Last known marital status of the debtor. It can take the following values: 1 not married, 2 married
nacionalidad	Whether the debtor is Chilean or foreign. 1 means Chilean, 2 mean foreign
sexo	Gender of the debtor. M means male and F means Female
fecha_nacimiento	Birth date of the debtor
deud_t_deuda	If it a loan shared with another institution
deud_e_deuda	Debt statement
deud_monto	Total loan amount
deud_monto_cuota_fija	Fixed installment amount
deud_fecha_exigibilidad	Date of enforceability of the loan
deud_numero_cuotas_fija	Number of fixed installments
paga_t_pagare	Type of promissory note. 1 means normal promissory note
paga_e_pagare	Promissory note statement. 1 means valid
monto_total_pagare	Total promissory note amount
conteo_pagare	Total promissory note count
num_declaraciones	Total income declarations counts
dejo_declarar	Whether the debtor stopped declaring their income or not. 0 means they keep declaring their income, 1 means they stopped
anio_ult_declaracion	Year of the last income declaration presented
dein_c_afp	Pension fund administrator code
dein_estado_civil	Marital status of the debtor in their last income declaration. 1 means not married, 2 means married
dein_hijo	Whether the debtor has children or not. 0 means they do not have children, 1 means that they have children
dein_c_inst_conyuge_deud	Institution code of the spouse of the debtor
dein_c_afp_conyuge	Pension fund administrator of the spouse of the debtor
dein_total_deudor_2	Total declared income of the debtor (in UTM)
dein_total_conyuge_2	Total declared income of the spouse of the debtor (in UTM)
deuda_conyuge	Whether the spouse of the debtor has a FSCU loan or not. 0 means they do not have a loan, 1 means they have.
anio_primera_declaracion	Year of the first income declaration of the debtor
proporcion_ingreso_cuota	Ratio of the average income of the debtor to the average installment amount
prom_proporcion_ingreso_deuda	Average ratio of the declared income of the debtor to the total amount of debt
std_proporcion_ingreso_deuda	Standard deviation of the ratio of the declared income of the debtor to the total amount of debt
dif_ingresos_declarados	Difference of declared income between the first and last income declaration of the debtor
prom_deudor	Average declared income of the debtor
std_deudor	Standard deviation of the declared income of the debtor
prom_conyuge	Average declared income of the spouse of the debtor
std_conyuge	Standard deviation of the declared income of the spouse of the debtor
cantidad_cambios_estado_civil	Number of changes in the marital status of the debtor through their income declarations
cuot_saldo	Balance of the last installment of the loan
cuot_e_cuota	Status of the last installment of the loan
avg_valor_cuota	Average effective installment amount
conteo_cuota	Number of effective installments
prom_cuota	Average installment amount
std_cuota	Standard deviation of the installments amounts
bole_monto_deuda_prom	Average sub-installment amount
bole_count_prom	Average number of sub-installments per installment
conteo_boletin	Number of sub-installments
matr_anio	Year of the last college enrollment of the debtor
matr_c_carrera	Code of the degree program covered by the loan
matr_e_matricula	Status of the last college enrollment of the debtor. 1 means the enrollment has a valid status
carr_t_carrera	Type of degree program in the last college enrollment of the debtor. 1 means undergraduate program
matr_c_institucion	Institution code in the last college enrollment of the debtor
conteo_matr	Total amount of enrollments of the debtor within the degree program covered by the loan
facultad	Faculty of the degree program
escuela	School of the degree program
steam	Whether the degree program covered by the loan is a STEM one or not. 1 means the degree program is a STEM program, 0 means it is not

**Table 5:** Dimensionality of the initial feature space by feature group prior to preprocessing.

Feature Group	No. of Features	Description
Demographic attributes	3	Static borrower characteristics such as sex, nationality, marital status, and family indicators.
Academic attributes	9	Variables related to enrollment history, degree program, faculty, school, and academic continuity.
Financial attributes	18	Loan-related variables including debt amounts, installments, balances, and repayment exposure.
Income declaration attributes	21	Variables derived from income declaration records, including counts, amounts, and status indicators.
Temporal and behavioral attributes	2	Aggregated and longitudinal indicators capturing variability, trends, and temporal dynamics of compliance.
Total	52	Initial dimensionality before preprocessing and feature selection.

**Table 6:** Summary of the dataset after preprocessing.

Characteristic	Value
Number of observations	8948
Initial number of features before preprocessing	52
Features after preprocessing	76
Numerical features	13
Binary features	5
Categorical features (one-hot encoded)	56
Date-derived features	2

**Table 7:** Baseline metrics.

Baseline	KNN	LightGBM	RandomForest
Precision minority class	0.559	1	0.881
Precision majority class	0.960	0.997	0.971
Recall minority class	0.320	0.951	0.505
Recall majority class	0.985	1	0.996
F-Score minority class	0.407	0.975	0.642
F-Score majority class	0.972	0.999	0.983

**Table 8:** Average training and inference time (seconds) for baseline classifiers using the full 76-feature space.

Baseline Runtime	KNN	LightGBM	RandomForest
Training	1.103	3.481	4.380
Inference	0.081	0.002	0.013

**Table 9:** Applied practical threshold metrics.

Practical Threshold	Precision	Recall	F1-Score
Minority Class	0.063	1	0.118
Majority Class	1	0.087	0.160

**Table 10:** KNN baseline comparison against Mutual Information filtering and L1 feature selection.

KNN	Recall Minority Class	Feature Count	Runtime (Seconds)
Base	0.320	76	1.1
Mutual Information	0.408	44	3.16
L1 Regularization	0.320	64	35.54

**Table 11:** LGBM baseline comparison against Mutual Information filtering and L1 feature selection.

LGBM	Recall Minority Class	Feature Count	Runtime (Seconds)
Base	0.951	76	3.48
Mutual Information	0.728	45	4.81
L1 Regularization	0.951	65	37.63

**Table 12:** Random Forest baseline comparison against Mutual Information filtering and L1 feature selection.

Random Forest	Recall Minority Class	Feature Count	Runtime (Seconds)
Base	0.505	76	4.38
Mutual Information	0.592	44	6.12
L1 Regularization	0.524	64	38.92

**Table 13:** Recall minority class.

MH	KNN			LightGBM			RandomForest		
	**Best**	**Avg**	**Std**	**Best**	**Avg**	**Std**	**Best**	**Avg**	**Std**
GWO	0.913	0.849	0.107	0.961	0.937	0.015	0.99	0.932	0.024
PSO	0.68	0.493	0.067	0.951	0.935	0.013	0.989	0.955	0.028
WOA	0.709	0.472	0.143	0.971	0.943	0.015	0.913	0.699	0.111

**Table 14:** F-Score minority class.

MH	KNN			LightGBM			RandomForest		
	**Best**	**Avg**	**Std**	**Best**	**Avg**	**Std**	**Best**	**Avg**	**Std**
GWO	0.94	0.874	0.104	0.975	0.954	0.009	0.986	0.943	0.015
PSO	0.753	0.551	0.064	0.975	0.957	0.01	0.946	0.886	0.064
WOA	0.772	0.508	0.133	0.975	0.964	0.011	0.922	0.779	0.061

**Table 15:** Precision minority class.

MH	KNN			LightGBM			RandomForest		
	**Best**	**Avg**	**Std**	**Best**	**Avg**	**Std**	**Best**	**Avg**	**Std**
GWO	0.989	0.903	0.105	0.99	0.972	0.014	0.99	0.955	0.024
PSO	0.843	0.627	0.067	1.0	0.982	0.013	0.989	0.955	0.028
WOA	0.849	0.578	0.161	1.0	0.986	0.015	0.985	0.896	0.05

**Table 16:** Recall majority class.

MH	KNN			LightGBM			RandomForest		
	**Best**	**Avg**	**Std**	**Best**	**Avg**	**Std**	**Best**	**Avg**	**Std**
GWO	0.999	0.994	0.006	0.999	0.998	0.001	0.999	0.997	0.002
PSO	0.992	0.982	0.004	1.0	0.999	0.001	0.999	0.998	0.001
WOA	1.0	0.978	0.018	1.0	0.999	0.001	0.999	0.995	0.004

**Table 17:** F-Score majority class.

MH	KNN			LightGBM			RandomForest		
	**Best**	**Avg**	**Std**	**Best**	**Avg**	**Std**	**Best**	**Avg**	**Std**
GWO	0.996	0.993	0.006	0.999	0.997	0.001	0.999	0.997	0.001
PSO	0.986	0.976	0.003	0.999	0.997	0.001	0.997	0.994	0.003
WOA	0.987	0.973	0.008	0.999	0.998	0.001	0.995	0.988	0.003

**Table 18:** Precision majority class.

MH	KNN			LightGBM			RandomForest		
	**Best**	**Avg**	**Std**	**Best**	**Avg**	**Std**	**Best**	**Avg**	**Std**
GWO	0.995	0.991	0.006	0.998	0.996	0.001	0.999	0.996	0.001
PSO	0.981	0.969	0.004	0.997	0.996	0.001	0.996	0.99	0.005
WOA	0.982	0.968	0.008	0.998	0.997	0.001	0.995	0.982	0.007

**Table 19:** Average number of selected features per optimization run across metaheuristics and classifiers.

Metaheuristic	KNN	RF	LightGBM	Average Per MH
GWO	9.8	13.7	24.0	15.9
PSO	33.1	28.6	36.3	32.7
WOA	24.8	24.4	50.8	33.3
Average per classifier	22.6	22.2	37.0	27.3

**Table 20:** Average offline optimization time (in seconds) for wrapper-based feature selection across metaheuristic–classifier configurations. Standard deviation computed across independent runs.

MH	Classifier	Avg. Optimization Time (s)	Std
PSO	KNN	616.66	14.74
WOA	KNN	493.50	69.27
GWO	KNN	475.14	43.09
PSO	LightGBM	1192.65	107.57
WOA	LightGBM	1482.66	234.15
GWO	LightGBM	909.22	80.00
PSO	RandomForest	2255.41	60.35
WOA	RandomForest	2115.94	270.84
GWO	RandomForest	2004.22	29.97

**Table 21:** Comparative summary of minority-class recall improvements across baseline, traditional feature selection, and metaheuristic optimization.

Classifier	Method	Recall Minority Class	Variation vs. Base	Feature Count
KNN	Base	0.320	–	76
KNN	Mutual Information	0.408	+0.088	44
KNN	L1 Regularization	0.320	0	64
KNN	Wrapper with MH (best avg. For GWO)	0.849	+0.529	9.8
LightGBM	Base	0.951	−	76
LightGBM	Mutual Information	0.728	−0.223	45
LightGBM	L1 Regularization	0.951	0	65
LightGBM	Wrapper with MH (best avg. For GWO)	0.937	−0.014	24
RandomForest	Base	0.505	−	76
RandomForest	Mutual Information	0.592	+0.087	44
RandomForest	L1 Regularization	0.524	+0.019	64
RandomForest	Wrapper with MH (best avg. For GWO)	0.932	+0.427	13.7

**Table 22:** Neminyi post hoc test considering OF (Equation 3).

Algorithm	GWO_KNN	GWO_LGBM	GWO_RF	PSO_KNN	PSO_LGBM	PSO_RF	WOA_KNN	WOA_LGBM	WOA_RF
GWO_KNN	X	$1.34\times 10^{-03}$	–	$3.15\times 10^{-03}$	$4.44\times 10^{-04}$	–	$7.83\times 10^{-05}$	$2.34\times 10^{-06}$	–
GWO_LGBM	$1.34\times 10^{-03}$	X	–	$4.50\times 10^{-14}$	–	$2.92\times 10^{-04}$	$1.11\times 10^{-16}$	–	$1.39\times 10^{-07}$
GWO_RF	–	–	X	$1.20\times 10^{-09}$	–	–	$2.97\times 10^{-12}$	–	$2.12\times 10^{-04}$
PSO_KNN	$3.15\times 10^{-03}$	$4.50\times 10^{-14}$	$1.20\times 10^{-09}$	X	$5.44\times 10^{-15}$	$1.17\times 10^{-02}$	–	$<1\times 10^{-16}$	–
PSO_LGBM	$4.44\times 10^{-04}$	–	–	$5.44\times 10^{-15}$	X	$8.77\times 10^{-05}$	$<1\times 10^{-16}$	-	$2.88\times 10^{-08}$
PSO_RF	–	$2.92\times 10^{-04}$	–	$1.17\times 10^{-02}$	$8.77\times 10^{-05}$	X	$4.00\times 10^{-04}$	$3.19\times 10^{-07}$	–
WOA_KNN	$7.83\times 10^{-05}$	$1.11\times 10^{-16}$	$2.97\times 10^{-12}$	–	$<1\times 10^{-16}$	$4.00\times 10^{-04}$	X	$<1\times 10^{-16}$	–
WOA_LGBM	$2.34\times 10^{-06}$	–	–	$<1\times 10^{-16}$	–	$3.19\times 10^{-07}$	$<1\times 10^{-16}$	X	$2.37\times 10^{-11}$
WOA_RF	–	$1.39\times 10^{-07}$	$2.12\times 10^{-04}$	–	$2.88\times 10^{-08}$	–	–	$2.37\times 10^{-11}$	X

Note: “X” denotes self-comparison. “–” indicates no statistically significant difference. Values correspond to *p*-values from the Neményi post hoc test.

**Table 23:** Summary of wins based on the Wilcoxon signed-rank test.

	Win
GWO_KNN	2
GWO_LGBM	5
GWO_RF	3
PSO_KNN	0
PSO_LGBM	5
PSO_RF	2
WOA_KNN	0
WOA_LGBM	5
WOA_RF	0

**Table 25:** Most parsimonious top-performing subset (GWO + LightGBM, Run 5). Encoded variables grouped by underlying administrative construct and data source.

Idx	Encoded Variable	Underlying Administrative Variable	Administrative Domain
4	deud_e_deuda_-1	deud_e_deuda (debt status)	Loan status registry
6	deud_e_deuda_2	deud_e_deuda (debt status)	Loan status registry
7	facultad_0	facultad (faculty of enrollment)	Academic records
15	facultad_8	facultad (faculty of enrollment)	Academic records
24	dein_c_afp_56	dein_c_afp (pension fund code)	Income declaration form
26	dein_c_afp_67	dein_c_afp (pension fund code)	Income declaration form
32	anio_ult_declaracion_2013	anio_ult_declaracion	Income declaration history
44	anio_primera_declaracion_2000	anio_primera_declaracion	Income declaration history
48	anio_primera_declaracion_2012	anio_primera_declaracion	Income declaration history
58	anio_primera_declaracion_2022	anio_primera_declaracion	Income declaration history
59	anio_primera_declaracion_2023	anio_primera_declaracion	Income declaration history
61	num_declaraciones	num_declaraciones	Declaration registry aggregation
70	conteo_cuota	conteo_cuota	Installment payment registry
75	anio_exigibilidad	anio_exigibilidad	Loan contract metadata

**Table 26:** Average inference time (seconds) for classifiers trained on metaheuristic-selected feature subsets.

Inference Runtime (s)	KNN	LightGBM	RandomForest
PSO	0.0672	0.0021	0.0422
GWO	0.0671	0.0029	0.0422
WOA	0.0747	0.0026	0.0419

**Figure A1:**
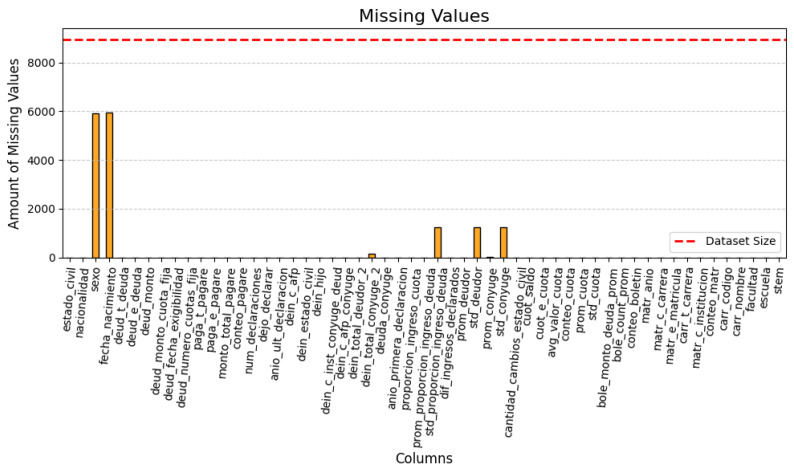
Distribution of missing values across features prior to preprocessing. The red dotted line indicates the total number of observations in the dataset.

**Figure A2:**
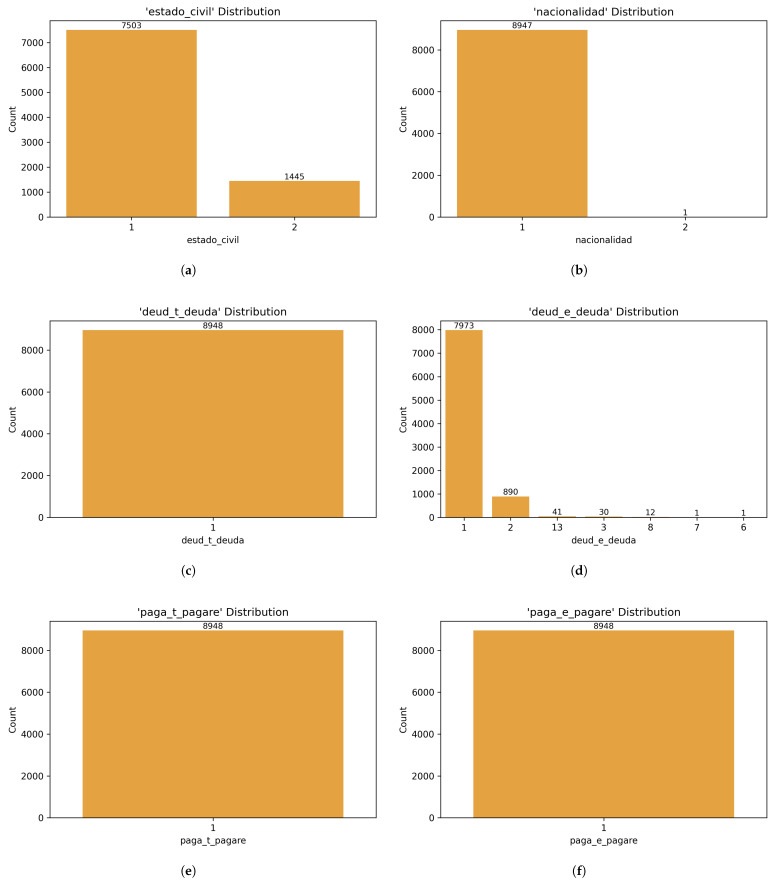
Frequency distributions of selected categorical variables (Part I). (**a**) Marital status (estado_civil); (**b**) nationality (nacionalidad); (**c**) debt type (deud_t_deuda); (**d**) debt status (deud_e_deuda); (**e**) promissory note type (paga_t_pagare); (**f**) promissory note status (paga_e_pagare).

**Figure A3:**
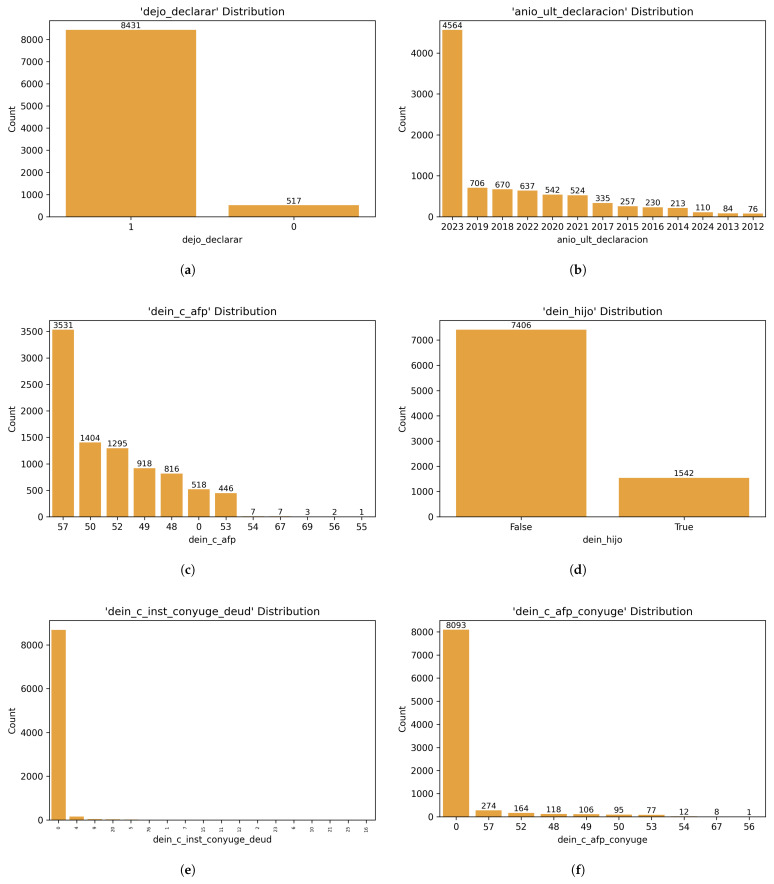
Frequency distributions of selected categorical variables (Part II). (**a**) Stopped declaring (dejo_declarar); (**b**) last declaration year (anio_ult_declaracion); (**c**) pension fund administrator (dein_c_afp); (**d**) has children (dein_hijo); (**e**) spouse institution code (dein_c_inst_conyuge_deud); (**f**) spouse pension fund administrator (dein_c_afp_conyuge).

**Figure A4:**
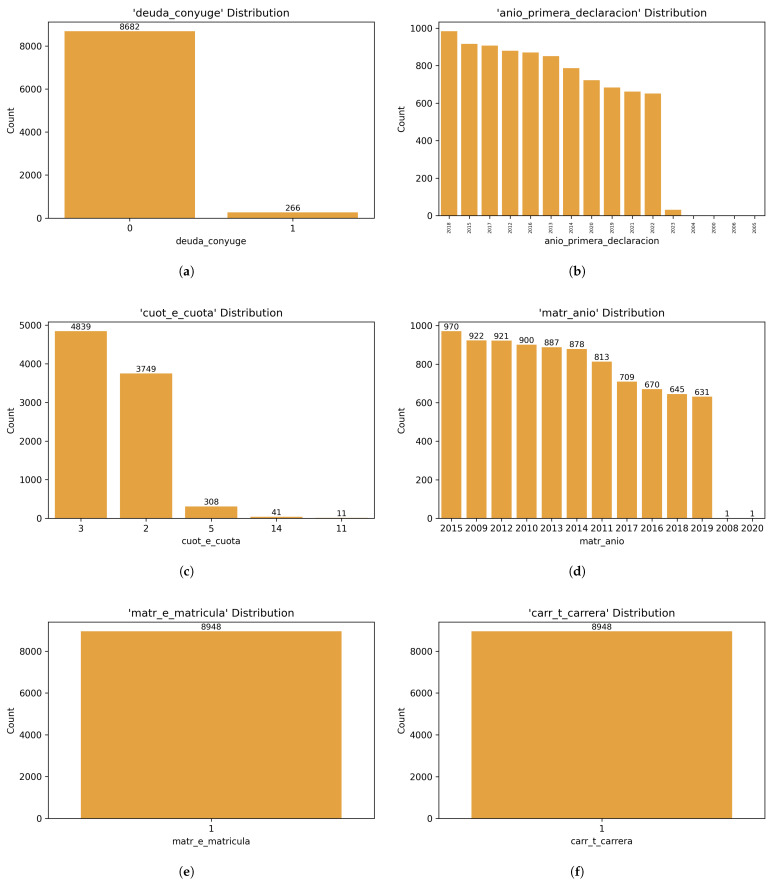
Frequency distributions of selected categorical variables (Part III). (**a**) Spouse has loan (deuda_conyuge); (**b**) first declaration year (anio_primera_declaracion); (**c**) installment status (cuot_e_cuota); (**d**) enrollment year (matr_anio); (**e**) enrollment status (matr_e_matricula); (**f**) program type (carr_t_carrera).

**Figure A5:**
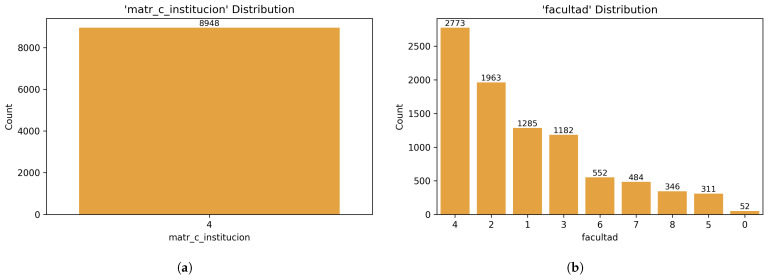
Frequency distributions of selected categorical variables (Part IV). (**a**) Institution code (matr_c_institucion); (**b**) faculty (facultad).

**Figure A6:**
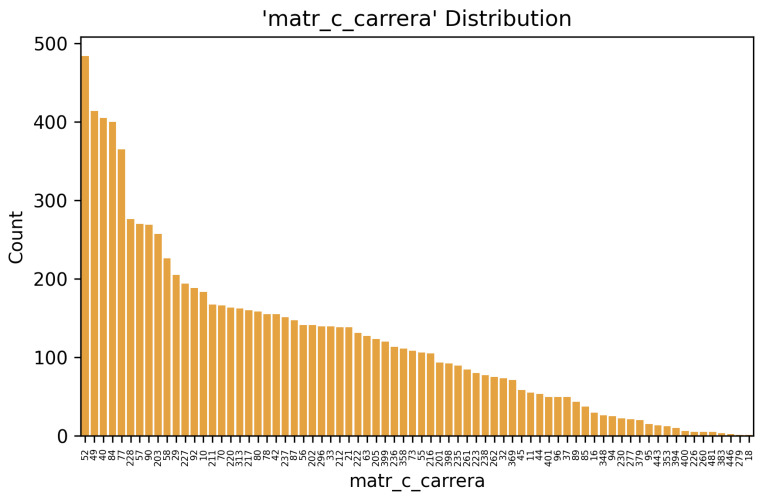
Distribution of degree program categories (matr_c_carrera) prior to consolidation.

**Figure A7:**
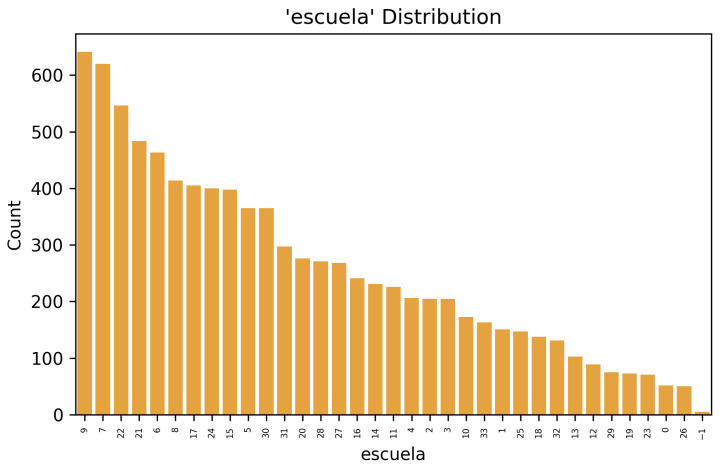
Distribution of school categories (escuela) prior to consolidation.

**Figure A8:**
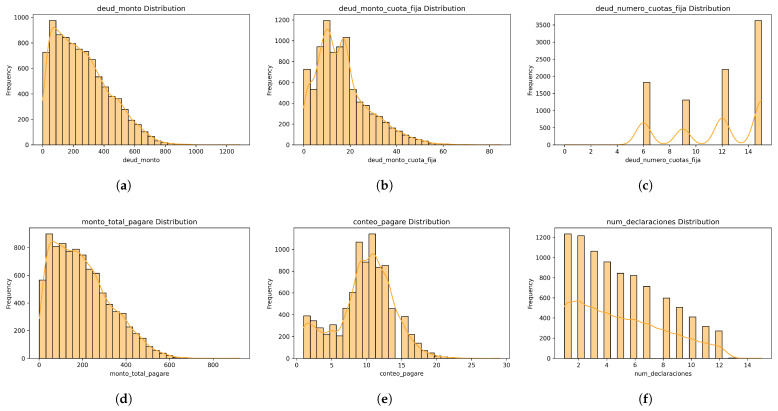
Distributions of representative numerical variables (Part I). (**a**) Total debt amount (deud_monto); (**b**) fixed installment amount (deud_monto_cuota_fija); (**c**) number of fixed installments (deud_numero_cuotas_fija); (**d**) total promissory note amount (monto_total_pagare); (**e**) promissory note count (conteo_pagare); (**f**) income declaration count (num_declaraciones). Histograms are overlaid with kernel density estimate (KDE) curves.

**Figure A9:**
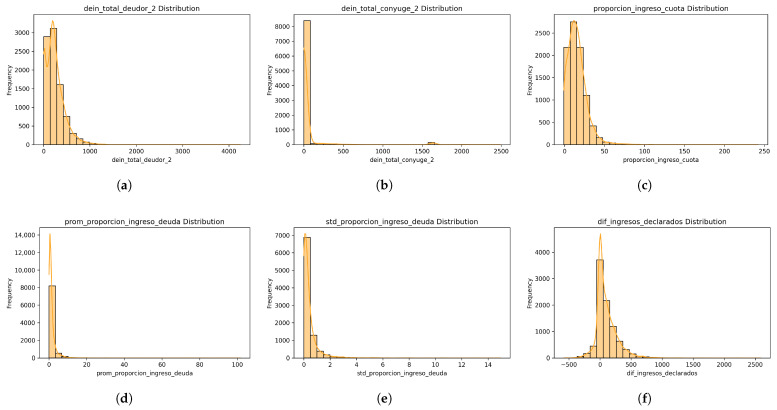
Distributions of representative numerical variables (Part II). (**a**) Declared debtor income (dein_total_deudor_2); (**b**) declared spouse income (dein_total_conyuge_2); (**c**) income-to-installment ratio (proporcion_ingreso_cuota); (**d**) average income-to-debt ratio (prom_proporcion_ ingreso_deuda); (**e**) std. income-to-debt ratio (std_proporcion_ingreso_deuda); (**f**) income difference (dif_ingresos_declarados). Histograms are overlaid with kernel density estimate (KDE) curves.

**Figure A10:**
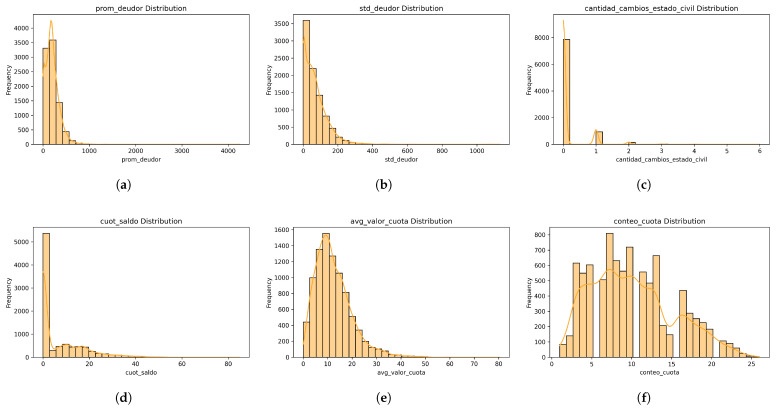
Distributions of representative numerical variables (Part III). (**a**) Average debtor income (prom_deudor); (**b**) std. debtor income (std_deudor); (**c**) marital status changes (cantidad_cambios_estado_civil); (**d**) installment balance (cuot_saldo); (**e**) average installment value (avg_valor_cuota); (**f**) effective installment count (conteo_cuota). Histograms are overlaid with kernel density estimate (KDE) curves.

**Figure A11:**
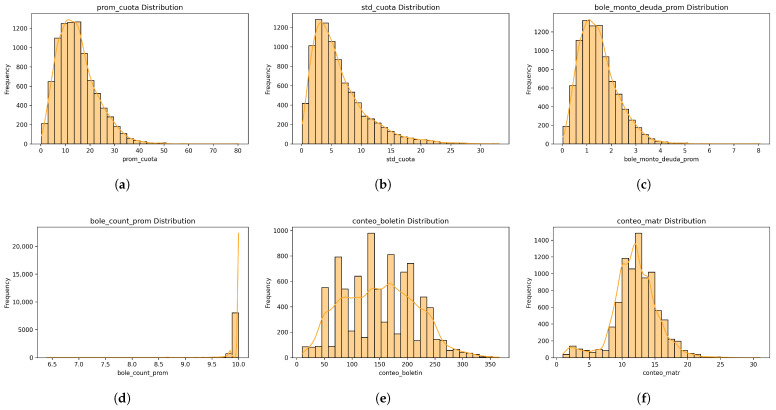
Distributions of representative numerical variables (Part IV). (**a**) Average installment amount (prom_cuota); (**b**) std. installment amount (std_cuota); (**c**) avg. bulletin debt amount (bole_monto_deuda_prom); (**d**) avg. bulletin count (bole_count_prom); (**e**) bulletin count (conteo_boletin); (**f**) enrollment count (conteo_matr). Histograms are overlaid with kernel density estimate (KDE) curves.

**Figure A12:**
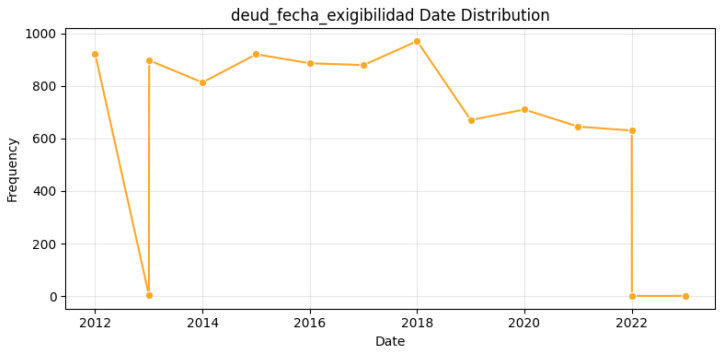
Distribution of enforceability dates associated with the loan obligations.

**Figure A13:**
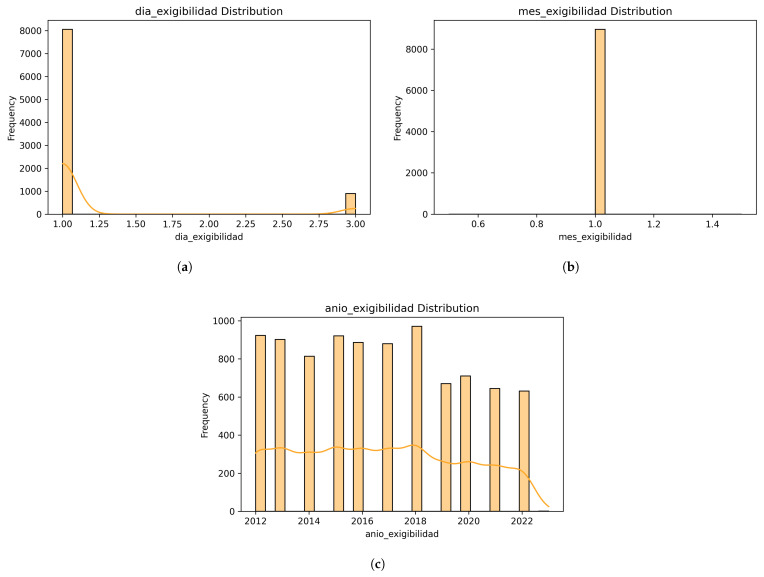
Decomposition of enforceability dates into day, month, and year components. (**a**) Day (dia_exigibilidad); (**b**) month (mes_exigibilidad); (**c**) year (anio_exigibilidad). Histograms are overlaid with kernel density estimate (KDE) curves.

## Data Availability

The dataset used in this study originates from internal institutional records and is subject to administrative and regulatory restrictions under institutional data governance policies. For transparency and verification purposes, the raw data and associated preprocessing code were provided to the journal during submission for editorial and peer-review evaluation. Due to these institutional constraints, the dataset cannot be made publicly available at this stage. The authors are preparing an anonymized and documented version of the dataset and intend to deposit it in an appropriate public repository once the necessary institutional approvals are obtained.

## Appendix A. Data Cleaning and Preprocessing: Supplementary Material

### Appendix A.1. Missing Data Diagnostics

**Table A1:** Features affected by missing values prior to data cleaning.

Feature	Missing Values
sexo	5925
fecha_nacimiento	5949
dein_total_conyuge_2	144
std_proporcion_ingreso_deuda	1233
std_deudor	1233
prom_conyuge	11
std_conyuge	1237

### Appendix A.5. Final Preprocessed Dataset

**Table A2:** Final dataset after pre-processing. The increase in dimensionality is due to the expansion of categorical variables via one-hot encoding.

Index	Feature	Index	Feature
0	estado_civil	38	anio_ult_declaracion_2019
1	dein_hijo	39	anio_ult_declaracion_2020
2	deuda_conyuge	40	anio_ult_declaracion_2021
3	stem	41	anio_ult_declaracion_2022
4	deud_e_deuda_-1	42	anio_ult_declaracion_2023
5	deud_e_deuda_1	43	anio_ult_declaracion_2024
6	deud_e_deuda_2	44	anio_primera_declaracion_2000
7	facultad_0	45	anio_primera_declaracion_2004
8	facultad_1	46	anio_primera_declaracion_2005
9	facultad_2	47	anio_primera_declaracion_2006
10	facultad_3	48	anio_primera_declaracion_2012
11	facultad_4	49	anio_primera_declaracion_2013
12	facultad_5	50	anio_primera_declaracion_2014
13	facultad_6	51	anio_primera_declaracion_2015
14	facultad_7	52	anio_primera_declaracion_2016
15	facultad_8	53	anio_primera_declaracion_2017
16	dein_c_afp_0	54	anio_primera_declaracion_2018
17	dein_c_afp_48	55	anio_primera_declaracion_2019
18	dein_c_afp_49	56	anio_primera_declaracion_2020
19	dein_c_afp_50	57	anio_primera_declaracion_2021
20	dein_c_afp_52	58	anio_primera_declaracion_2022
21	dein_c_afp_53	59	anio_primera_declaracion_2023
22	dein_c_afp_54	60	deud_monto
23	dein_c_afp_55	61	num_declaraciones
24	dein_c_afp_56	62	dein_total_deudor_2
25	dein_c_afp_57	63	dein_total_conyuge_2
26	dein_c_afp_67	64	proporcion_ingreso_cuota
27	dein_c_afp_69	65	std_proporcion_ingreso_deuda
28	cuot_e_cuota_-1	66	std_deudor
29	cuot_e_cuota_2	67	cantidad_cambios_estado_civil
30	cuot_e_cuota_3	68	cuot_saldo
31	anio_ult_declaracion_2012	69	avg_valor_cuota
32	anio_ult_declaracion_2013	70	conteo_cuota
33	anio_ult_declaracion_2014	71	bole_count_prom
34	anio_ult_declaracion_2015	72	conteo_matr
35	anio_ult_declaracion_2016	73	mes_exigibilidad
36	anio_ult_declaracion_2017	74	dia_exigibilidad
37	anio_ult_declaracion_2018	75	anio_exigibilidad
